# The risks of adverse events with mirtazapine for adults with major depressive disorder: a systematic review with meta-analysis and trial sequential analysis

**DOI:** 10.1186/s12888-024-06396-6

**Published:** 2025-01-22

**Authors:** Caroline Barkholt Kamp, Johanne Juul Petersen, Pascal Faltermeier, Sophie Juul, Christina Dam Bjerregaard Sillassen, Faiza Siddiqui, Rebecca Kjaer Andersen, Joanna Moncrieff, Mark Abie Horowitz, Michael Pascal Hengartner, Irving Kirsch, Christian Gluud, Janus Christian Jakobsen

**Affiliations:** 1 Copenhagen Trial Unit, Centre for Clinical Intervention Research, The Capital Region, Copenhagen University Hospital – Rigshospitalet, Blegdamsvej 9, Copenhagen Ø, DK-2100 Denmark; 2 Department of Regional Health Research, The Faculty of Health Sciences, University of Southern Denmark, Odense, DK-5000 Denmark; 3 MSH Medical School Hamburg, University of Applied Sciences and Medical University, 20457 Hamburg, Germany; 4 Stolpegaard Psychotherapy Centre, Mental Health Services in the Capital Region of Denmark, Gentofte, DK-2820 Denmark; 5 Department of Psychology, University of Copenhagen, Copenhagen, Denmark; 6 Department of Cardiology and Endocrinology, Slagelse Hospital, Region of Zealand, Slagelse, DK-4200 Denmark; 7 Division of Psychiatry (This position is honorary for MAH), University College London, London, W1T 7BN UK; 8 Research and Development Department, North East London NHS Foundation Trust (NELFT), London, RM13 8EU UK; 9 Department of Applied Psychology, Zurich University of Applied Sciences, Zurich, 8005 Switzerland; 10 Program in Placebo Studies, Harvard Medical School, Boston, MA 02215 USA

**Keywords:** Antidepressants, Mirtazapine, Major depressive disorder, Adverse events, Systematic review

## Abstract

**Background:**

Mirtazapine is used to treat depression worldwide, and the effects of mirtazapine on depression rating scales are well-known. Our primary objective was to assess the risks of adverse events with mirtazapine for major depressive disorder.

**Methods:**

We searched relevant sources from inception to 7 March 2024 for randomised clinical trials comparing mirtazapine versus placebo in adults with major depressive disorder. The primary outcomes were suicides or suicide attempts, serious adverse events, and non-serious adverse events. Data were synthesised using meta-analysis and Trial Sequential Analysis.

**Results:**

We included 17 trials randomising 2,131 participants to mirtazapine versus placebo. All results were at high risk of bias, and the certainty of the evidence was very low. The included trials assessed outcomes at a maximum of 12 weeks after randomisation. Meta-analysis and Trial Sequential Analysis showed insufficient information to determine the effects of mirtazapine on the risks of suicides or suicide attempts and serious adverse events. Meta-analyses showed that mirtazapine increased the risks of somnolence, weight gain, dry mouth, dizziness, and increased appetite but decreased the risk of headaches.

**Conclusions:**

There is a lack of evidence on the effects of mirtazapine on suicides and serious adverse events. Mirtazapine increases the risks of somnolence, weight gain, dry mouth, dizziness, and increased appetite. Mirtazapine might decrease the risk of headaches. The long-term effects of mirtazapine are unknown.

**Prospero id:**

CRD42022315395.

**Supplementary Information:**

The online version contains supplementary material available at 10.1186/s12888-024-06396-6.

## Background

Major depressive disorder is a psychiatric disorder associated with an increased risk of suicidal ideation and behaviour, reduced quality of life, and impaired cognition [1–6]. Globally, approximately 280 million people are affected by major depressive disorder, causing a severe burden on patients and societies [7]. Mirtazapine is an antidepressant that acts as a presynaptic α_2_-receptor antagonist in the central nervous system. Mirtazapine is thought to inhibit negative feedback mechanisms, leading to increased levels of noradrenaline and serotonin in the synaptic cleft, which enhances the postsynaptic availability of noradrenaline and serotonin. Additionally, mirtazapine is thought to block the postsynaptic 5-HT_2_ and 5-HT_3_ receptors (serotonergic receptors), selectively enhancing 5-HT_1A_-mediated serotonergic transmission [8, 9]. Mirtazapine is also a potent antagonist of the histamine receptor (H_1_) which is hypothesised to result in sedative effects and weight gain, mainly appearing at lower doses [10]. Mirtazapine is commonly used as a first- or second-line treatment of major depressive disorder and often for older adults [11–14]. Mirtazapine is used for treatment of major depressive disorder in many countries, including several EU countries, the United States, and the United Kingdom [15–17]. According to the U.S. Food & Drug Administration (FDA) and the European Medicines Agency (EMA), the recommended doses are 15–45 mg once daily [18, 19].

The effects of mirtazapine on depression rating scale scores are well-known [20–23]. Previous reviews have not systematically assessed the risks of all adverse events with mirtazapine compared to placebo [22–29]. Our primary objective was to assess the risks of adverse events with mirtazapine versus placebo in the treatment of adults with major depressive disorder. Our secondary objective was to assess the beneficial effects of mirtazapine.

## Methods

Our protocol predefined the methodology based on the recommendations of the Cochrane Handbook of Systematic Reviews of Interventions [30, 31]. The protocol was also preregistered in the PROSPERO database (ID: CRD42022315395). We report this systematic review based on the Preferred Reporting Items for Systematic Reviews and Meta-Analysis (PRISMA) guidelines (Supplementary Text 1) [32, 33]. This systematic review is a part of a larger project investigating the effects of all antidepressants for major depressive disorder [34–38].

### Search strategy

An information specialist searched the Cochrane Central Register of Controlled Trials (CENTRAL), Medical Literature Analysis and Retrieval System Online (MEDLINE), Excerpta Medica Database (Embase), Latin American and Caribbean Health Sciences Literature (LILACS), PsycINFO, Science Citation Index Expanded (SCI-EXPANDED), Social Sciences Citation Index (SSCI), Conference Proceedings Citation Index—Science (CPCI-S), and Conference Proceedings Citation Index—Social Science & Humanities (CPCI-SSH) to identify relevant trials. We searched all databases from their inception to 7 March 2024. For a detailed search strategy for all electronic databases, see Supplementary Text 2. We searched clinical trial registers, websites of pharmaceutical companies, and websites of medicines agencies to identify unpublished data (Supplementary Text 3). We requested clinical study reports from FDA, EMA, and national medicines agencies (Supplementary Text 3).

### Selection criteria

Our eligibility criteria included trials randomising adults with a primary diagnosis of major depressive disorder as defined by standardised diagnostic criteria, such as the Diagnostic and Statistical Manual of Mental Disorders [39] or the International Classification of Diseases [40]. The experimental intervention was mirtazapine, and the control interventions were either placebo, ‘active placebo’, or ‘no intervention’.

### Data extraction and risk of bias assessment

Four review authors (CBK, SJ, CDBS, and FS) independently screened abstracts and articles to identify relevant trials. Five review authors (JJP, PF, RKA, CDBS, and CBK) independently extracted data and assessed the risks of bias based on the Cochrane Risk of Bias tool, version 2 (RoB 2) [41, 42]. Any discrepancies were resolved through internal discussion or, if required, through discussion with the senior author (JCJ).

### Outcomes and subgroup analyses

The primary outcomes were suicides or suicide attempts, serious adverse events (based on the International Council on Harmonisation of technical requirements for registration of pharmaceuticals for human use—Good Clinical Practice (ICH-GCP) definition of a serious adverse event, which is any untoward medical occurrence that resulted in death, was life-threatening, required hospitalisation or prolonging of existing hospitalisation and resulted in persistent or significant disability or jeopardised the participant [43]), and non-serious adverse events. Exploratory outcomes were depressive symptoms measured on the 17-item Hamilton Depression Rating Scale (HDRS-17), quality of life, all adverse events, suicidal ideation, level of functioning, depressive symptoms measured on the Montgomery-Asberg Depression Rating Scale (MADRS) [44], the Beck’s Depression Inventory (BDI) [45], or HDRS-6 [46, 47], withdrawal symptoms, and proportion of participants that guessed their treatment allocation. Short-term follow-up (defined as the assessment in the trial closest to three months after randomisation) was of primary interest. Secondly, long-term follow-up (defined as the assessment in the trial closest to six months after randomisation) was assessed. We also planned several subgroup analyses [31].

### Assessment of statistical and clinical significance

We performed meta-analyses according to the recommendations of the Cochrane Handbook for Systematic Reviews of Interventions [41], Keus et al. [48], and the eight-step procedure by Jakobsen et al. [49]. We adjusted the threshold for statistical significance by the number of primary outcomes according to Jakobsen et al. [49] and therefore used a *p*-value of 0.025 or less as threshold. We analysed data using the software Stata version 17 [50]. We used both random-effects (Hartung-Knapp-Sidik-Jonkman) [51] and fixed-effect model meta-analyses (Mantel-Haenszel for dichotomous outcomes and inverse variance for continuous outcomes) to assess intervention effects [41, 52]. We primarily reported the most conservative result (highest *p*-value) and considered the less conservative result a sensitivity analysis [49]. We adjusted for zero-event cells using treatment-arm continuity correction in analyses of suicides or suicide attempts and serious adverse events. Trial Sequential Analysis was used to control for random errors by estimating the diversity-adjusted required information size (DARIS) [53–61]. We used Grading Recommendations Assessment Development Evaluation (GRADE) to assess the certainty of evidence [62–64].

## Results

**Fig. 1:**
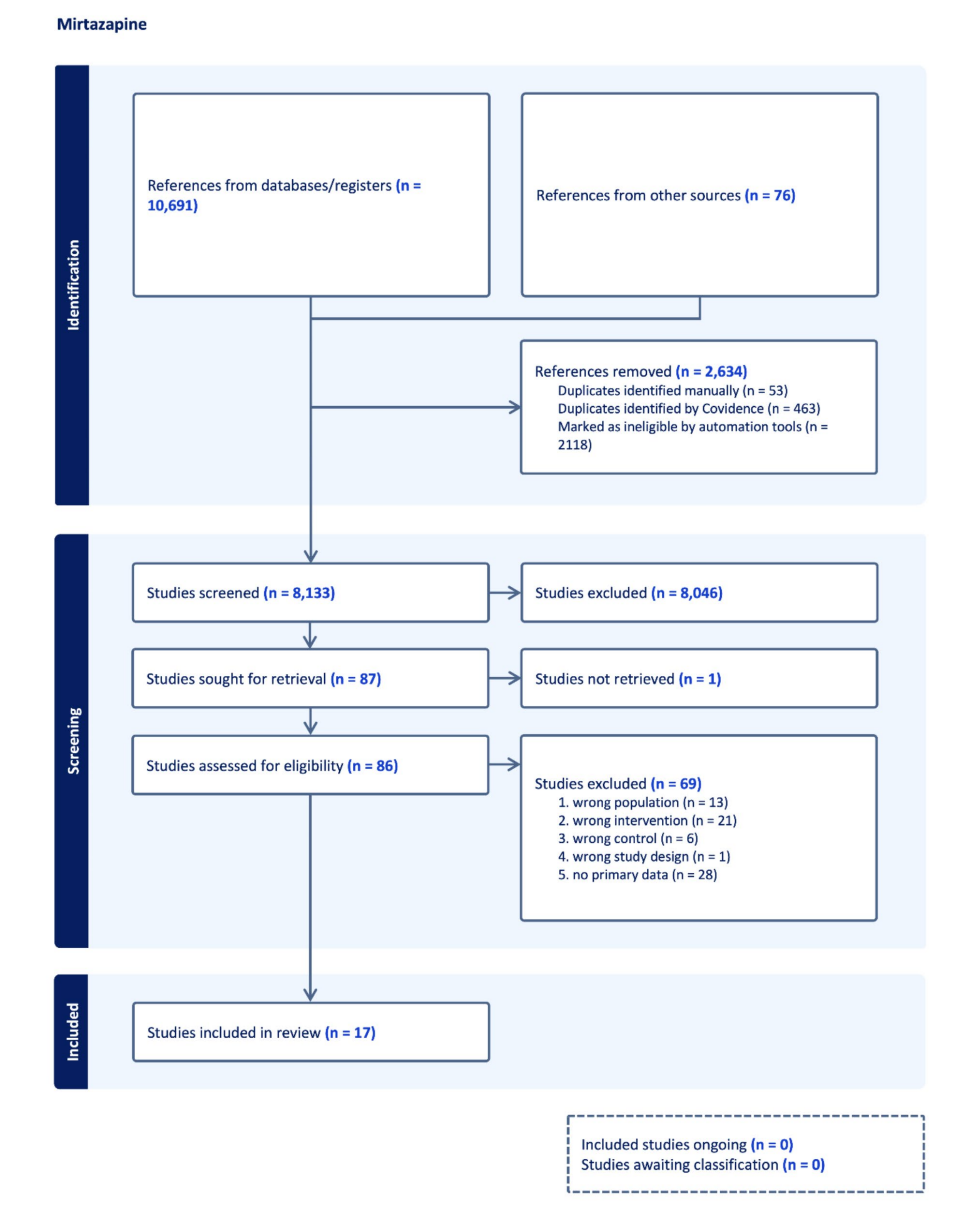
PRISMA flow diagram

A total of 17 trials randomising 2,131 participants to mirtazapine versus placebo were included [65–90] (Fig. 1). No trials compared mirtazapine with ‘active placebo’ or no intervention. We identified unpublished data for six trials [68, 81, 87–90]. All trials included both men and women aged 18 years or older with a primary diagnosis of major depressive disorder, while one trial only included participants aged 55 years or older (Supplementary Table 1) [70]. The mean HDRS baseline scores ranged from 20.9 to 31.5 (Supplementary Table 1). The included trials assessed outcomes at a maximum of 12 weeks after randomisation. Most trials did not adequately report the proportion of participants with missing data at follow-up, and it was, therefore, not possible to perform ‘best-worst/worst-best’ sensitivity analyses. All trials were assessed at overall high risk of bias (Supplementary Fig. 1).

### Primary outcomes

#### Suicides or suicide attempts

**Fig. 2:**
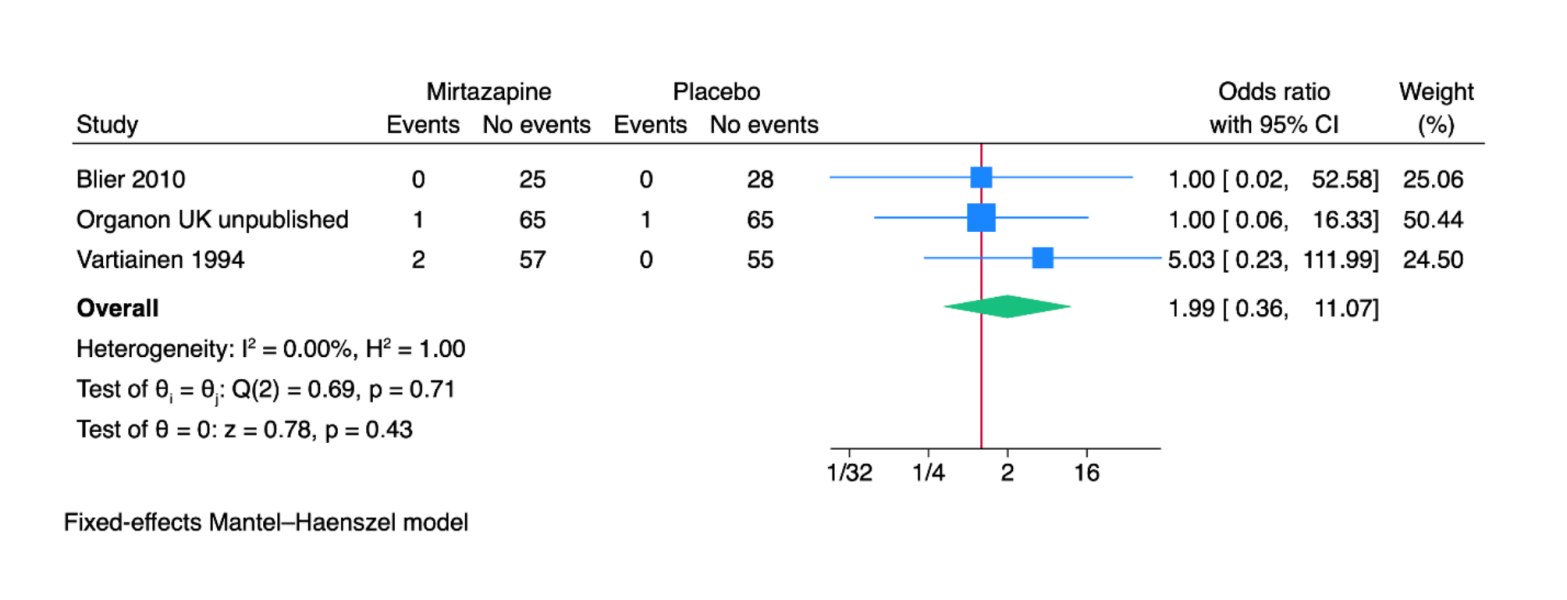
Meta-analysis of mirtazapine versus placebo on suicides or suicide attempts

Only three of 17 trials reported results on suicides or suicide attempts [69, 79, 90]. The included trials only assessed outcomes at the end of the treatment period, i.e. from five to six weeks after randomisation. A total of 3/150 (2.0%) experimental participants attempted or committed suicide versus 1/149 (0.7%) control participants. Meta-analysis showed no evidence of a difference (odds ratio (OR) 1.99; 95% confidence interval (CI) 0.36 to 11.07; *p* = 0.43; 3 trials; Bayes factor: 1.26) (Fig. 2). Visual inspection of the forest plot and statistical tests (I^2^ = 0.0%) indicated no clear signs of heterogeneity. Trial Sequential Analysis showed that we did not have enough information to confirm or reject that mirtazapine influenced the risk of suicides or suicide attempts with a relative risk reduction of 20% (no graph produced). This outcome result was assessed as overall high risk of bias, and the certainty of the evidence was very low (Supplementary Table 2).

Tests of interaction comparing the effects of using a placebo washout period (*p* = 0.71) and co-interventions (*p* = 0.71) showed no evidence of differences (Supplementary Figs. 2–3). The remaining predefined subgroup analyses could not be performed due to a lack of relevant data.

#### Serious adverse events

**Fig. 3:**
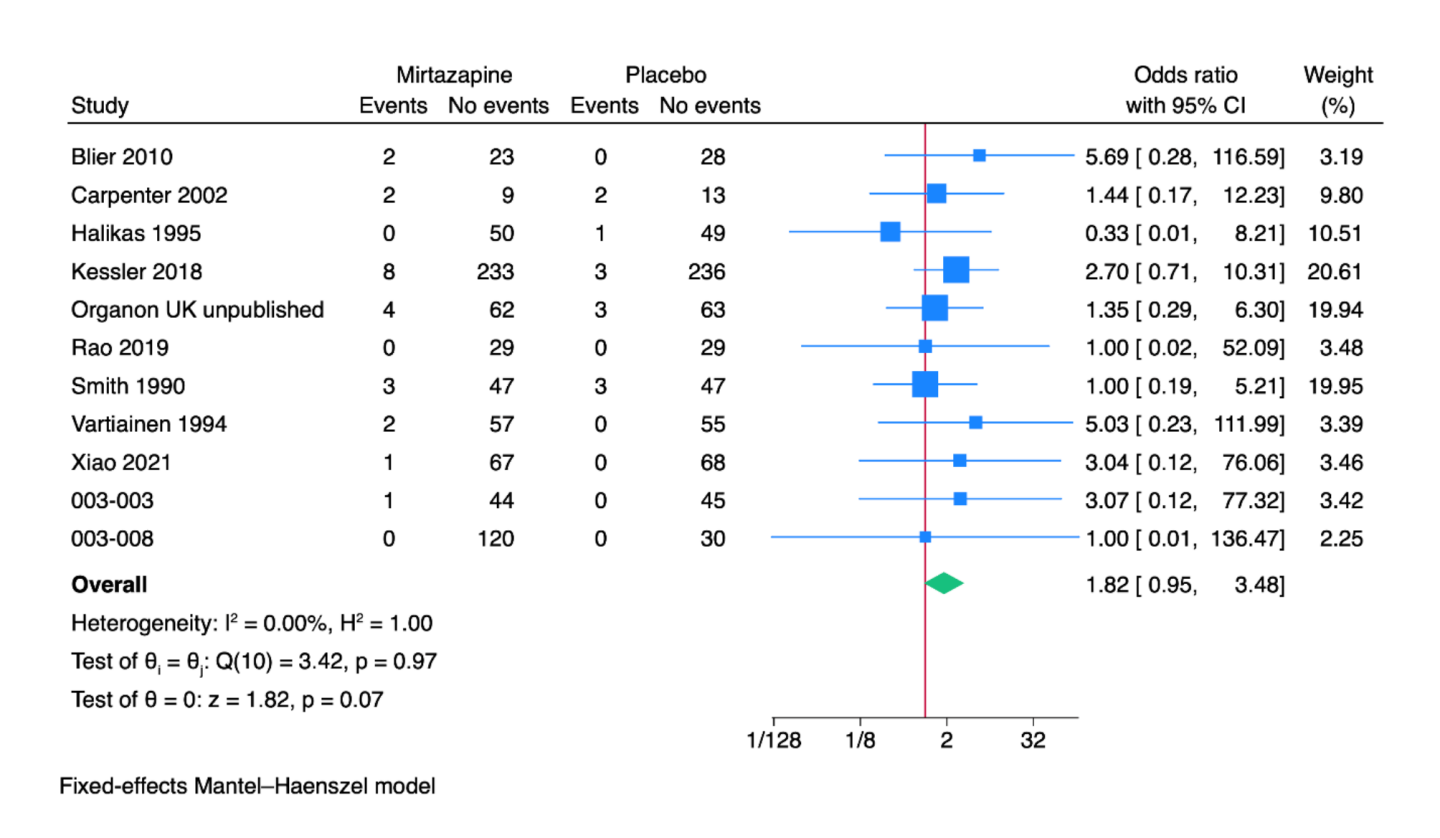
Meta-analysis of mirtazapine versus placebo on serious adverse events

Eleven trials reported results on serious adverse events [69–71, 79, 82–84, 86, 88–90]. The included trials only assessed outcomes at the end of the treatment period, i.e. from four to 12 weeks after randomisation. A total of 23/764 (3.0%) experimental participants experienced a serious adverse event versus 12/675 (1.8%) control participants. Meta-analysis showed no evidence of a difference (OR 1.82; 95% CI 0.95 to 3.48; *p* = 0.07; 11 trials; Bayes factor: 0.43) (Fig. 3). Visual inspection of the forest plot and statistical tests (I^2^ = 0.0%) indicated no clear signs of heterogeneity. Trial Sequential Analysis showed that we did not have enough information to confirm or reject that mirtazapine influenced the risk of serious adverse events with a relative risk reduction of 20% (no graph produced). This outcome result was assessed as overall high risk of bias, and the certainty of the evidence was very low (Supplementary Table 2).

Tests of interaction comparing the effects of using a placebo washout period (*p* = 0.38), co-interventions (*p* = 0.41), and risks of for-profit bias (*p* = 0.60) showed no evidence of differences (Supplementary Figs. 4–6). The remaining predefined subgroup analyses could not be performed due to a lack of relevant data.

The meta-analyses of each specific serious adverse event showed no evidence of differences (Supplementary Figs. 7–8, Supplementary Table 3).

#### Non-serious adverse events

**Fig. 4:**
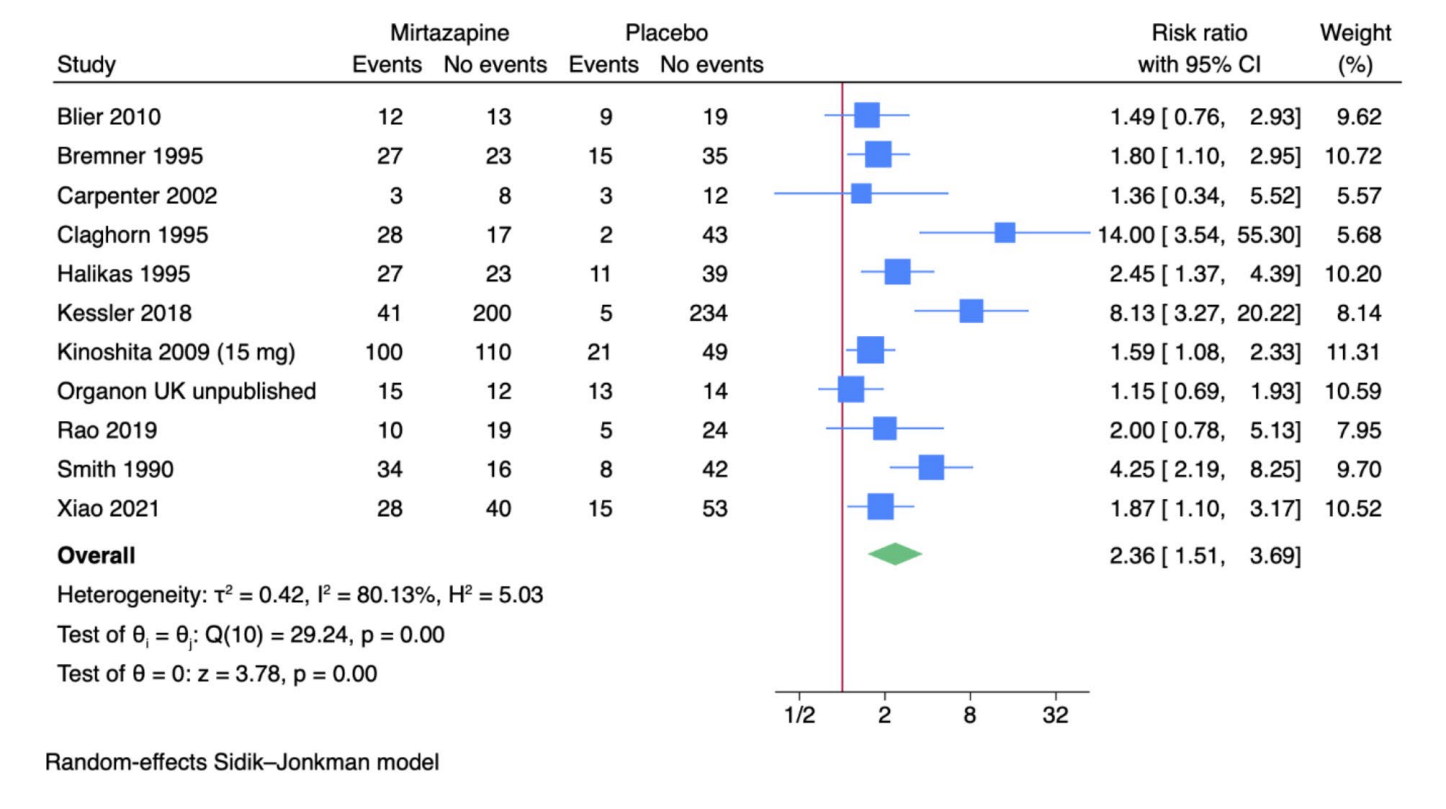
Meta-analysis of mirtazapine versus placebo on non-serious adverse events

Eleven trials reported results on non-serious adverse events [65, 66, 69–71, 73, 79, 82–84, 86]. The included trials only assessed outcomes at the end of the treatment period, i.e. from four to 12 weeks after randomisation. A total of 325/806 (40.3%) experimental participants experienced a non-serious adverse event compared with 107/671 (15.9%) control participants. Meta-analysis showed evidence of a harmful effect (risk ratio (RR) 2.36; 95% CI 1.51 to 3.69; *p* < 0.01; 11 trials; Bayes factor: 0.68) (Fig. 4). Visual inspection of the forest plot and statistical tests (I^2^ = 80.1%) indicated heterogeneity that could not be resolved. Trial Sequential Analysis showed that we did not have enough information to confirm or reject that mirtazapine influenced the risk of non-serious adverse events with a relative risk reduction of 20% (no graph produced). This outcome result was assessed as overall high risk of bias, and the certainty of the evidence was very low (Supplementary Table 2).

When each specific non-serious adverse event was analysed separately, 5/17 meta-analyses showed evidence of a harmful effect of mirtazapine: somnolence (RR 2.61; 95% CI 1.26 to 5.37; *p* = 0.01; I^2^ = 84.7%; 10 trials; NNH: 4), weight gain (RR 4.75; 95% CI 2.05 to 10.99; *p* < 0.01; I^2^ = 32.5%; 7 trials; NNH: 10), dry mouth (RR 1.57; 95% CI 1.02 to 2.42; *p* = 0.04; I^2^ = 55.2%; 9 trials; NNH: 11), dizziness (RR 2.54; 95% CI 1.18 to 5.45; *p* = 0.02; I^2^ = 17.3%; 6 trials; NNH: 19), and increased appetite (RR 3.22; 95% CI 1.34 to 7.73; *p* = 0.01; I^2^ = 14.1%; 7 trials; NNH: 26) (Supplementary Figs. 9–13). When each specific non-serious adverse event was analysed separately, 1/17 meta-analysis showed evidence of a beneficial effect of mirtazapine: headache (RR 0.54; 95% CI 0.35 to 0.83; *p* < 0.01; I^2^ = 14.4%; 8 trials; number needed to treat (NNT): 22) (Supplementary Fig. 14). The remaining meta-analyses showed no evidence of differences (Supplementary Figs. 15–25, Supplementary Table 4).

### Exploratory outcomes and sensitivity analyses

#### HDRS-17

Seven trials reported results on HDRS-17 [73, 76, 78, 79, 82, 83, 86]. The included trials only assessed outcomes at the end of the treatment period, i.e. from four to six weeks after randomisation. Meta-analysis showed evidence of a beneficial effect (mean difference (MD) − 2.77 HDRS-17 points; 95% CI − 3.94 to − 1.60; *p* < 0.01; 7 trials) (Supplementary Fig. 26), however, the effect size was below a proposed minimal important difference of three HDRS-17 points [20]. Visual inspection of the forest plot and statistical tests (I^2^ = 33.2%) indicated no clear signs of heterogeneity. This outcome result was assessed as overall high risk of bias.

#### Quality of life

Only two trials analysing a total of 455 participants reported results on quality of life [83, 84]. One trial reported a beneficial effect of mirtazapine on the Quality of Life and Satisfaction Questionnaire subscores four weeks after randomisation: physical health (F(1,17) = 7.38; *p =* 0.02), general activities (F(1,15) = 6.31; *p* = 0.02), and leisure activities (F(1,17) = 6.47; *p* = 0.02) [83]. The other trial reported no evidence of a difference on EQ-5D-5 L 12 weeks after randomisation (adjusted MD 0.01; 95% CI − 0.02 to 0.05; *p* = 0.40) [84].

#### MADRS, BDI, and HDRS-6

Six trials reported results on MADRS, BDI or HDRS-6 [71, 72, 76, 78, 79, 84, 86]. The included trials only assessed outcomes at the end of the treatment period, i.e. four to 12 weeks after randomisation. Meta-analysis showed evidence of a beneficial effect (standardised mean difference (SMD) − 0.40; 95% CI − 0.75 to − 0.06; *p* = 0.02; 6 trials) (Supplementary Fig. 27), however, the effect size was below a proposed minimal important difference of a 0.5 SMD [20]. Visual inspection of the forest plot and statistical tests (I^2^ = 80.7%) indicated heterogeneity. This outcome result was assessed as overall high risk of bias.

#### Level of functioning

Only two trials analysing a total of 444 participants reported results on level of functioning [83, 84]. One trial reported a beneficial effect of mirtazapine on the Global Assessment of Functioning scale four weeks after randomisation (F(1,23) = 4.87; *p* = 0.04) [83]. The other trial reported a beneficial effect of mirtazapine on the Short Form 12 mental functioning subscore (adjusted MD 3.91; 95% CI 1.63 to 6.20; *p* < 0.01), but no evidence of a difference on the physical functioning subscore (adjusted MD − 1.09; 95% CI − 2.75 to 0.57; *p* = 0.20) 12 weeks after randomisation [84].

#### Remaining results

Due to a lack of relevant data, it was not possible to analyse the remaining exploratory outcomes, i.e. suicidal ideation, withdrawal symptoms, and the proportion of participants who guessed their treatment allocation. It was also not possible to assess any outcomes at a long-term follow-up defined as six months or more after randomisation. We performed all meta-analyses as both fixed-effect and random-effects meta-analyses and primarily reported the most conservative results as the main results. For the less conservative results, please see Supplementary Figs. 28–51.

## Discussion

We conducted a systematic review assessing the risks of adverse events with mirtazapine for adults with major depressive disorder. We included 17 trials randomising 2,131 participants to mirtazapine versus placebo. All results were at high risk of bias, and the certainty of the evidence was very low, particularly due to poor reporting, lack of data, lack of information on blinding [91], missing outcome data, and inappropriate analysis methods. The included trials assessed outcomes at a maximum of 12 weeks after randomisation. Meta-analysis and Trial Sequential Analysis showed insufficient information to assess the effects of mirtazapine on the risks of suicides or suicide attempts and serious adverse events. Meta-analyses showed that mirtazapine increased the risks of somnolence, weight gain, dry mouth, dizziness, and increased appetite but decreased the risk of headaches. Exploratory meta-analyses confirmed the well-known statistically significant effects of antidepressants on depression rating scales, but effect sizes were just below proposed minimal important differences (three HDRS-17 points or 0.5 SMD [20]). Due to a lack of relevant data, it was not possible to assess the effects of mirtazapine on suicidal ideation, withdrawal symptoms, the proportion of participants who guessed their treatment allocation, or any outcomes at a long-term follow-up. The evidence for the overall benefits of mirtazapine is inadequate, and there is no evidence that pertains to long-term treatment. Patients need to be informed about the inadequacy of evidence before initiating treatment with mirtazapine.

We did not assess response and remission primarily because the focus of the present review was to assess adverse effects. Moreover, response and remission have several limitations: (1) the assessments of remission and response are usually based on single HDRS scores, raising concerns about whether these scores truly reflect full remission or adequate response to the intervention; (2) transforming continuous data into dichotomous data results in information loss, and results can be heavily influenced by data distribution and the selection of arbitrary cut-points [30, 92–94]; (3) when participants cross the arbitrary threshold for remission (often HDRS below 7–12) or response (often 50% HDRS reduction), the actual change in HDRS scores might still be minimal [95]; (4) focusing solely on how many participants reach a predefined threshold for benefit overlooks those who may be deteriorating at the same time. For example, if results show substantial benefits of mirtazapine in terms of remission and response rates but very small average effects (as our findings indicate), this could suggest that similar proportions of participants experience an increase in HDRS compared to placebo.

Our systematic review has various strengths. It is the first systematic review to systematically assess all adverse effects of mirtazapine for adults with major depressive disorder. Access to data on adverse effects is crucial for enabling both patients and clinicians to make well-informed decisions regarding treatment options. The predefined methodology was based on the Cochrane Handbook for Systematic Reviews of Interventions [96], PRISMA [33], Trial Sequential Analysis [53, 59], the eight-step procedure by Jakobsen et al. [49], the GRADE approach [62], and risks of systematic and random errors, external validity, publication bias, and heterogeneity were taken into account. Furthermore, unpublished data were included in the analyses to increase the validity of our results [96–99].

Our systematic review highlighted important limitations of the available data. First, the included trials only reported results at the end of treatment at a maximum of 12 weeks, leaving the long-term effects of mirtazapine unknown. Half of patients on antidepressants in the United Kingdom and 70% of patients in the USA have used them for more than two years [100, 101]. There is an urgent need for trials with long-term follow-up to assess the benefits and harms of mirtazapine. This is particularly pertinent for medications that are associated with tolerance and withdrawal effects, which tend to show diminishing effects over time [102]. Second, all included trials were assessed at overall high risk of bias particularly driven by risks of bias due to poor reporting, lack of blinding of outcome assessors, missing outcome data, and inappropriate analysis methods. Studies have shown that trials at high risk of bias tend to overestimate the beneficial effects and underestimate the harmful effects of the experimental intervention [103–112], so our present results may be biased in the same direction. The reporting and assessments of adverse events were especially inadequate. Third, the certainty of the evidence was very low for all outcome results. It is, therefore, possible that trials conducted with higher methodological quality will show different results. Fourth, only three of the included trials reported on suicides or suicide attempts. Notably, we identified two suicides in unpublished data of a trial that were not included in the published report [72]. The lack of data on suicides and suicide attempts is particularly problematic given the association between major depressive disorder and increased risks of suicidal behaviour [1, 4, 5]. There is a need for larger trials at low risk of bias to assess the risks of suicides and suicide attempts. Fifth, only two trials had publicly available protocols or trial registrations, further compromising the scientific validity of the available trial results. Sixth, it was impossible to draw meaningful conclusions regarding the effects of different dosages due to highly heterogeneous and limited data, high risk of bias, and evidence of very low certainty. Additionally, dosages were inconsistently reported as means, maximums, or ranges (based on either the full treatment period or e.g. only last week of treatment), further complicating data analysis and interpretation.

Our systematic review also has limitations We included three primary outcomes, which increased the risk of type I errors. To control the risks of random errors, we adjusted our threshold for significance according to the number of primary outcomes, but we did not adjust the thresholds for significance according to the total number of comparisons, including exploratory outcomes and subgroup analyses. These limitations should be considered when interpreting our results.

## Conclusions

There is a lack of evidence on the effects of mirtazapine on suicides and serious adverse events. Mirtazapine increases the risks of somnolence, weight gain, dry mouth, dizziness, and increased appetite. Mirtazapine might decrease the risk of headaches. The long-term effects of mirtazapine are unknown.

## Electronic supplementary material

Below is the link to the electronic supplementary material.

Supplementary Material 1

## Data Availability

All data generated or analysed during this study are included in this published article and its supplementary information files.

## Abbreviations

BDI: Beck’s Depression Inventory
CENTRAL: Cochrane Central Register of Controlled Trials
CI: confidence interval
CPCI-S: Conference Proceedings Citation Index—Science
CPCI-SSH: Conference Proceedings Citation Index—Social Science & Humanities
DARIS: diversity-adjusted required information size
EMA: European Medicines Agency
EMBASE: Excerpta Medica Database
FDA: U.S. Food & Drug Administration
GRADE: Grading of Recommendations, Assessment, Development and Evaluation
HDRS: Hamilton Depression Rating Scale
LILACS: Latin American and Caribbean Health Sciences Literature
MADRS: Montgomery-Asberg Depression Rating Scale
MDs: mean differences
MEDLINE: Medical Literature Analysis and Retrieval System Online
NNH: number needed to harm
NNT: number needed to treat
OR: odds ratio
PRISMA: Preferred Reporting Items for Systematic Reviews and Meta-Analysis
RoB 2: Cochrane Risk of Bias tool – version 2
RRs: risk ratios
SCI-EXPANDED: Science Citation Index Expanded
SMD: standardised mean difference
SSCI: Social Sciences Citation Index
