# Supplementary material for: The risks of adverse events with mirtazapine for adults with major depressive disorder: a systematic review with meta-analysis and trial sequential analysis
Source: BMC Psychiatry. 2025 Jan 22;25:67. doi: 10.1186/s12888-024-06396-6 (PMC11755810; doi:10.1186/s12888-024-06396-6)
Supplement: Supplementary file 1 — Supplementary Material 1 [file 12888_2024_6396_MOESM1_ESM.pdf]

Supplementary material for:

The risks of adverse events with mirtazapine for adults with major depressive disorder: a systematic review with meta-analysis and Trial Sequential Analysis

CB Kamp, JJ Petersen, P Faltermeier, S Juul, CDB Sillassen, F Siddiqui, RK Andersen, J Moncrieff, MA Horowitz, MP Hengartner, I Kirsch, C Gluud, JC Jakobsen

Table of Contents

Supplementary Figure 1: Risk of Bias 2 assessments 4

Supplementary Figure 2: Subgroup analysis of placebo washout on suicides or suicide attempts 5

Supplementary Figure 3: Subgroup analysis of co-interventions on suicides or suicide attempts................................6

Supplementary Figure 4: Subgroup analysis of placebo washout on serious adverse events 7

Supplementary Figure 5: Subgroup analysis of co-interventions on serious adverse events...8

Supplementary Figure 6: Subgroup analysis of for-profit bias on serious adverse events 9

Supplementary Figure 7: Meta-analysis of mirtazapine versus placebo on sexual dysfunction.................................10

Supplementary Figure 8: Meta-analysis of mirtazapine versus placebo on anxiety...11

Supplementary Figure 9: Meta-analysis of mirtazapine versus placebo on somnolence 12

Supplementary Figure 10: Meta-analysis of mirtazapine versus placebo on weight gain 13

Supplementary Figure 11: Meta-analysis of mirtazapine versus placebo on dry mouth..14

Supplementary Figure 12: Meta-analysis of mirtazapine versus placebo on dizziness 15

Supplementary Figure 13: Meta-analysis of mirtazapine versus placebo on increased appetite...............................16

Supplementary Figure 14: Meta-analysis of mirtazapine versus placebo on headache...17

Supplementary Figure 15: Meta-analysis of mirtazapine versus placebo on amblyopia 18

Supplementary Figure 16: Meta-analysis of mirtazapine versus placebo on asthenia 19

Supplementary Figure 17: Meta-analysis of mirtazapine versus placebo on blurred vision.......................................20

Supplementary Figure 18: Meta-analysis of mirtazapine versus placebo on constipation..21

Supplementary Figure 19: Meta-analysis of mirtazapine versus placebo on dyspepsia 22

Supplementary Figure 20: Meta-analysis of mirtazapine versus placebo on insomnia..23

Supplementary Figure 21: Meta-analysis of mirtazapine versus placebo on nausea...24

Supplementary Figure 22: Meta-analysis of mirtazapine versus placebo on sedation 25

Supplementary Figure 23: Meta-analysis of mirtazapine versus placebo on stomach discomfort..............................26

Supplementary Figure 24: Meta-analysis of mirtazapine versus placebo on tachycardia..27

Supplementary Figure 25: Meta-analysis of mirtazapine versus placebo on tremor 28

Supplementary Figure 26: Meta-analysis of mirtazapine versus placebo on HDRS-17 29

Supplementary Figure 27: Meta-analysis of mirtazapine versus placebo on MADRS, BDI, and HDRS-6.....................30

Supplementary Figure 28: Meta-analysis of mirtazapine versus placebo on suicides or suicide attempts (sensitivity analysis) 31

Supplementary Figure 29: Meta-analysis of mirtazapine versus placebo on serious adverse events (sensitivity analysis) 32

Supplementary Figure 30: Meta-analysis of mirtazapine versus placebo on sexual dysfunction (sensitivity analysis)33

Supplementary Figure 31: Meta-analysis of mirtazapine versus placebo on anxiety (sensitivity analysis).................34

Supplementary Figure 32: Meta-analysis of mirtazapine versus placebo on non-serious adverse events (sensitivity analysis) 35

Supplementary Figure 33: Meta-analysis of mirtazapine versus placebo on somnolence (sensitivity analysis) 36

Supplementary Figure 34: Meta-analysis of mirtazapine versus placebo on weight gain (sensitivity analysis) 37

Supplementary Figure 35: Meta-analysis of mirtazapine versus placebo on dry mouth (sensitivity analysis)............38

Supplementary Figure 36: Meta-analysis of mirtazapine versus placebo on dizziness (sensitivity analysis) 39

Supplementary Figure 37: Meta-analysis of mirtazapine versus placebo on increased appetite (sensitivity analysis)40

Supplementary Figure 38: Meta-analysis of mirtazapine versus placebo on headache (sensitivity analysis).............41

Supplementary Figure 39: Meta-analysis of mirtazapine versus placebo on amblyopia (sensitivity analysis) 42

Supplementary Figure 40: Meta-analysis of mirtazapine versus placebo on asthenia (sensitivity analysis)................43

Supplementary Figure 41: Meta-analysis of mirtazapine versus placebo on blurred vision (sensitivity analysis)........44

Supplementary Figure 42: Meta-analysis of mirtazapine versus placebo on constipation (sensitivity analysis)..........45

Supplementary Figure 43: Meta-analysis of mirtazapine versus placebo on dyspepsia (sensitivity analysis).............46

Supplementary Figure 44: Meta-analysis of mirtazapine versus placebo on insomnia (sensitivity analysis)..............47

Supplementary Figure 45: Meta-analysis of mirtazapine versus placebo on nausea (sensitivity analysis).................48

Supplementary Figure 46: Meta-analysis of mirtazapine versus placebo on sedation (sensitivity analysis)................49

Supplementary Figure 47: Meta-analysis of mirtazapine versus placebo on stomach discomfort (sensitivity analysis) 50

Supplementary Figure 48: Meta-analysis of mirtazapine versus placebo on tachycardia (sensitivity analysis)...........51

Supplementary Figure 49: Meta-analysis of mirtazapine versus placebo on tremor (sensitivity analysis) 52

Supplementary Figure 50: Meta-analysis of mirtazapine versus placebo on HDRS-17 (sensitivity analysis) 53

Supplementary Figure 51: Meta-analysis of mirtazapine versus placebo on MADRS, BDI, and HDRS-6 (sensitivity analysis) 54

Supplementary Text 1: PRISMA checklist...55

Supplementary Text 2: Search strategy 58

Supplementary Text 3: Search strategy for unpublished data 61

Supplementary Table 1: Characteristics of the included trials...67

Supplementary Table 2: Summary of findings...69

Supplementary Table 3: Serious adverse events in the included trials 70

Supplementary Table 4: Individual non-serious adverse events..71

Supplementary Figure 1: Risk of Bias 2 assessments

Study	D1	D2	D3	D4	D5	Overall
Blier 2010	-	X	X	X	-	X
Bremner 1995	-	X	X	X	-	X
Carpenter 2002	-	X	X	X	-	X
Claghorn 1995	-	X	X	X	-	X
Halikas 1995	-	X	X	X	-	X
Kessler 2018	+	X	X	-	+	X
Kinoshita 2009	X	X	X	X	-	X
Organon UK unpublished	-	X	X	X	-	X
Rao 2019	-	X	X	X	-	X
Smith 1990	-	X	X	X	-	X
Vartiainen 1994	-	X	X	X	-	X
Xiao 2021	+	X	X	X	-	X
003-003	-	X	X	X	-	X
003-008	-	X	X	X	-	X

Domains:
D1: Bias arising from the randomization process.
D2: Bias due to deviations from intended intervention.
D3: Bias due to missing outcome data.
D4: Bias in measurement of the outcome.
D5: Bias in selection of the reported result.

Judgement
X High
- Some concerns
+ Low

Based on assessments of the primary outcomes.

Supplementary Figure 2: Subgroup analysis of placebo washout on suicides or suicide attempts

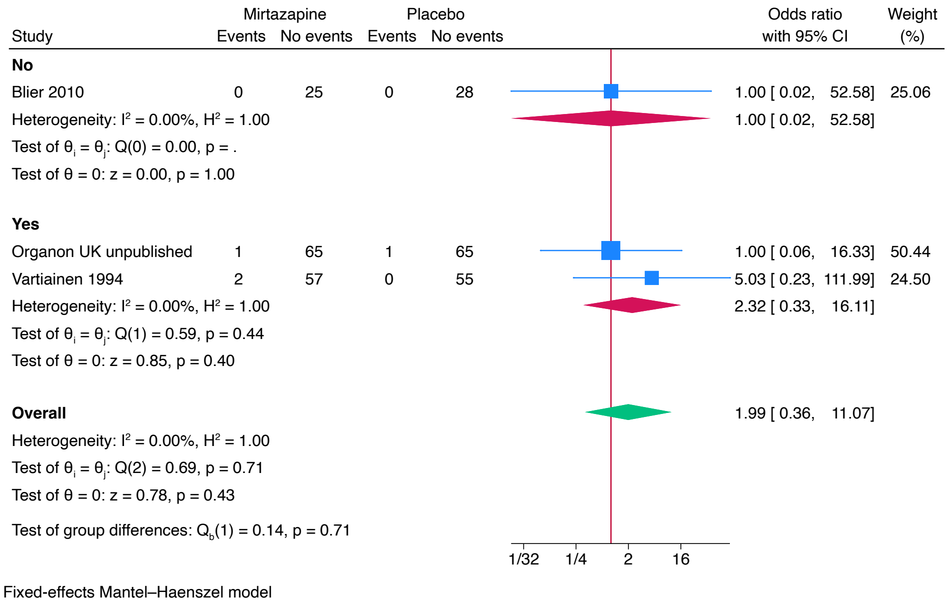

Supplementary Figure 3: Subgroup analysis of co-interventions on suicides or suicide attempts

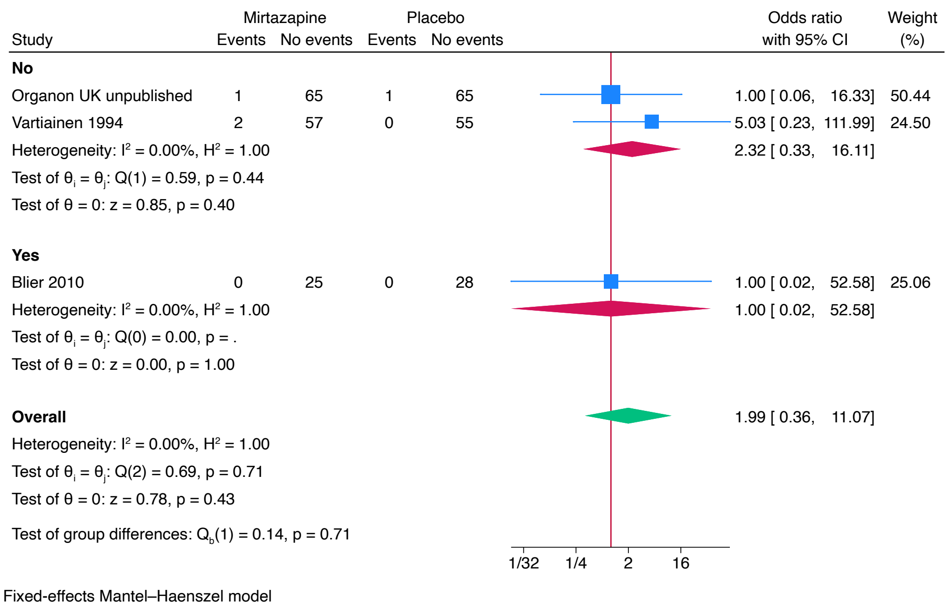

Supplementary Figure 4: Subgroup analysis of placebo washout on serious adverse events

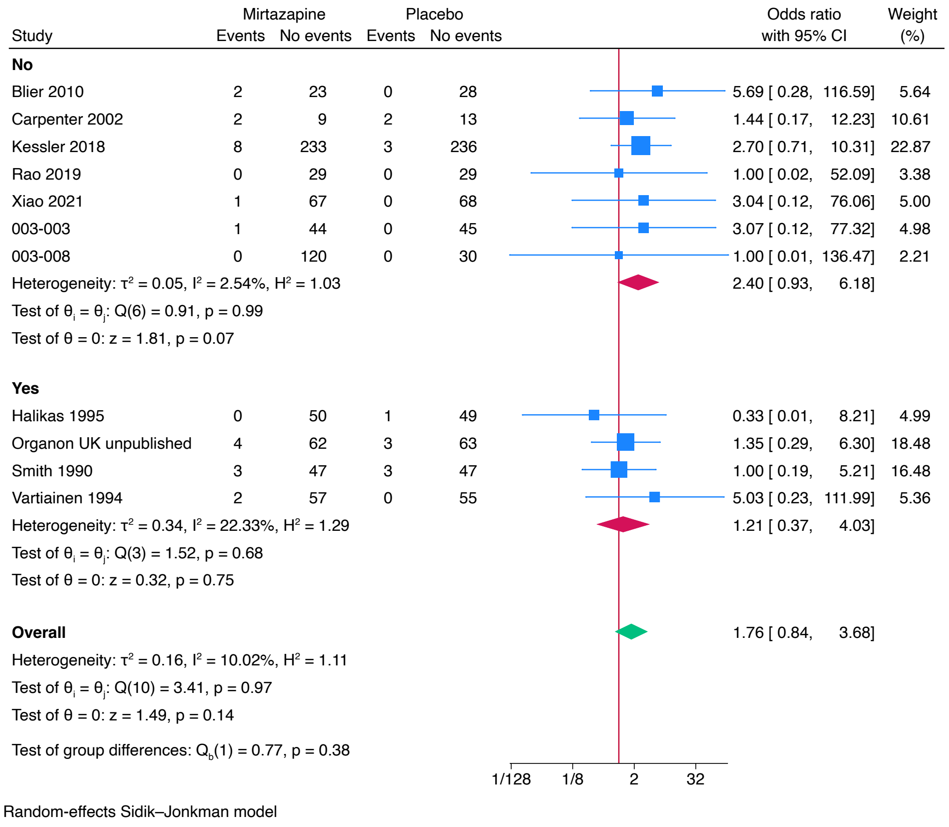

STATA

Supplementary Figure 5: Subgroup analysis of co-interventions on serious adverse events

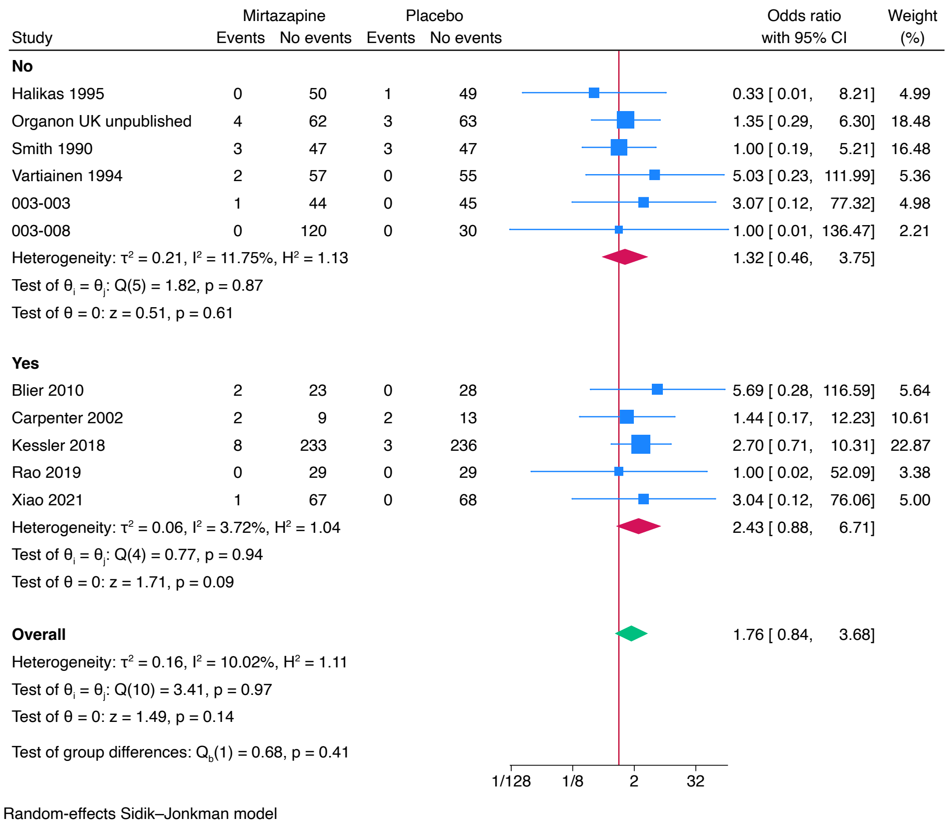

Supplementary Figure 6: Subgroup analysis of for-profit bias on serious adverse events

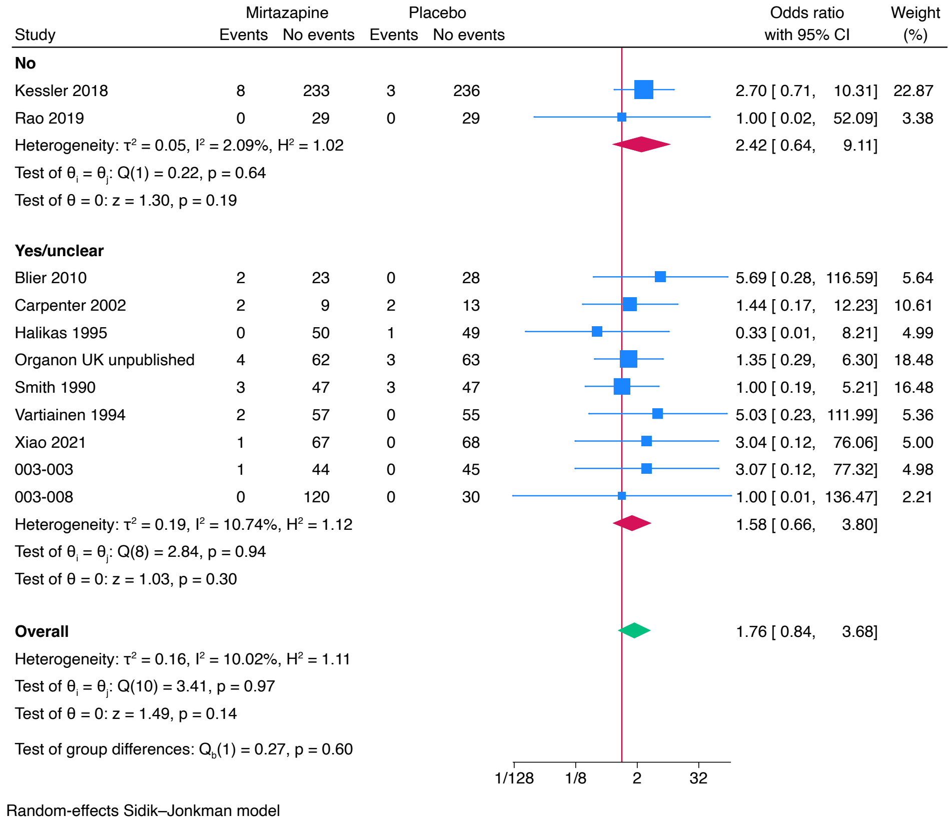

Supplementary Figure 7: Meta-analysis of mirtazapine versus placebo on sexual dysfunction

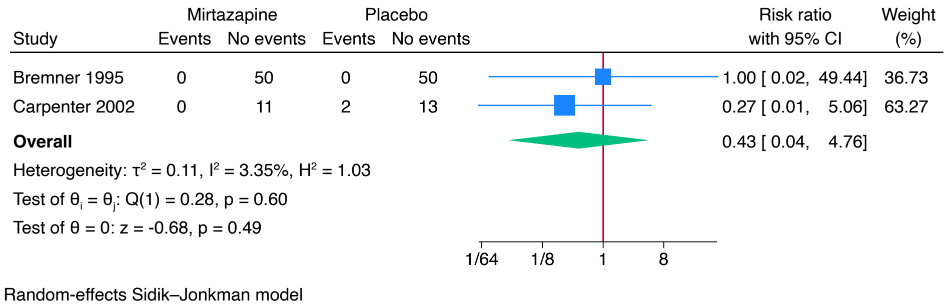

Supplementary Figure 8: Meta-analysis of mirtazapine versus placebo on anxiety

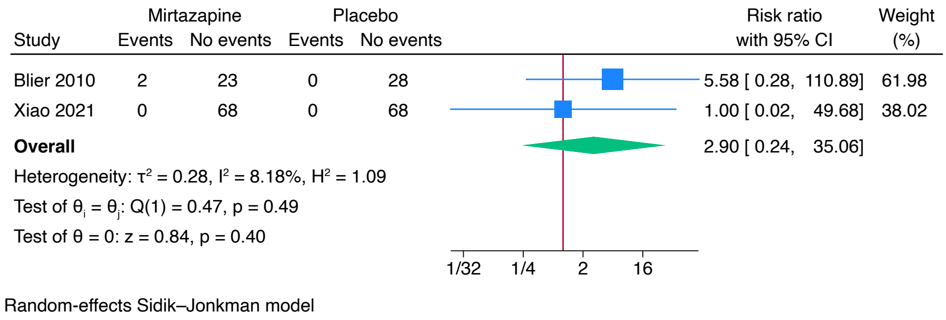

Supplementary Figure 9: Meta-analysis of mirtazapine versus placebo on somnolence

Study	Mirtazapine Events	Mirtazapine No events	Placebo Events	Placebo No events	Risk ratio with 95% CI	Weight (%)
Blier 2010	2	23	3	25	0.75 [0.14, 4.11]	7.95
Bremner 1995	23	27	11	39	2.09 [1.15, 3.82]	12.98
Carpenter 2002	1	10	2	13	0.68 [0.07, 6.61]	5.93
Claghorn 1995	28	17	2	43	14.00 [3.54, 55.30]	9.42
Halikas 1995	27	23	11	39	2.45 [1.37, 4.39]	13.06
Kessler - 2018	41	200	5	234	8.13 [3.27, 20.22]	11.63
Kinoshita - 2009	100	110	21	49	1.59 [1.08, 2.33]	13.72
Organon UK unpublished	2	62	4	55	0.46 [0.09, 2.42]	8.14
Smith 1990	34	16	8	42	4.25 [2.19, 8.25]	12.74
Xiao 2021	6	62	0	68	13.00 [0.75, 226.32]	4.42
**Overall**					2.61 [1.26, 5.37]	

Heterogeneity: $\tau^2 = 0.95$, $I^2 = 84.65\%$, $H^2 = 6.52$

Test of $\theta_i = \theta_j$: $Q(9) = 28.79$, $p = 0.00$

Test of $\theta = 0$: $z = 2.60$, $p = 0.01$

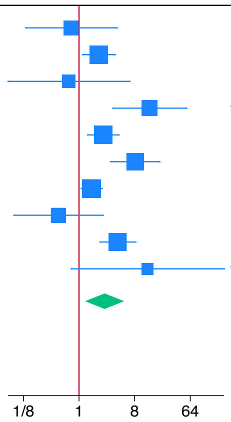

Random-effects Sidik–Jonkman model

Supplementary Figure 10: Meta-analysis of mirtazapine versus placebo on weight gain

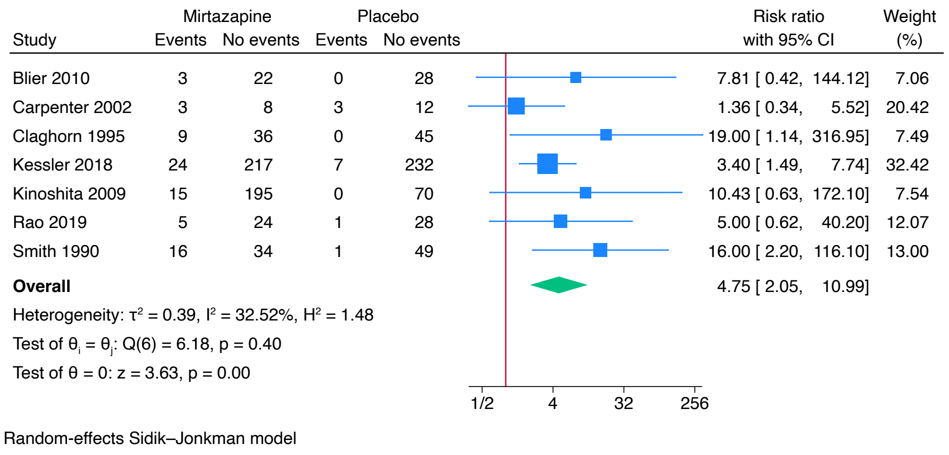

Supplementary Figure 11: Meta-analysis of mirtazapine versus placebo on dry mouth

Study	Mirtazapine Events	Mirtazapine No events	Placebo Events	Placebo No events	Risk ratio with 95% CI	Weight (%)
Blier 2010	12	13	9	19	1.49 [0.76, 2.93]	14.57
Bremner 1995	27	23	15	35	1.80 [1.10, 2.95]	17.43
Carpenter 2002	0	11	2	13	0.27 [0.01, 5.06]	1.97
Halikas 1995	24	26	6	44	4.00 [1.79, 8.94]	12.69
Kinoshita 2009	42	168	10	60	1.40 [0.74, 2.64]	15.20
Organon UK unpublished	6	58	6	53	0.92 [0.31, 2.70]	9.43
Rao 2019	10	19	5	24	2.00 [0.78, 5.13]	10.91
Smith 1990	9	41	10	40	0.90 [0.40, 2.02]	12.59
Xiao 2021	4	64	2	66	2.00 [0.38, 10.56]	5.21
**Overall**					1.57 [1.02, 2.42]	

Heterogeneity: $\tau^2 = 0.22$, $I^2 = 55.20\%$, $H^2 = 2.23$

Test of $\theta_i = \theta_j$: Q(8) = 10.06, p = 0.26

Test of $\theta = 0$: z = 2.05, p = 0.04

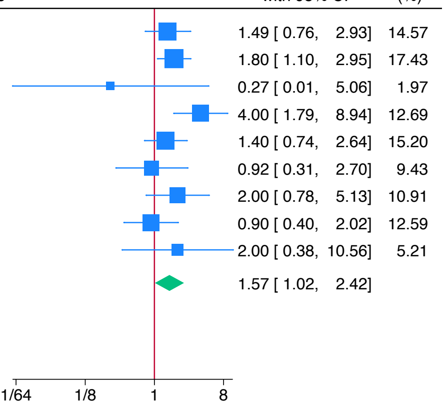

Random-effects Sidik–Jonkman model

Supplementary Figure 12: Meta-analysis of mirtazapine versus placebo on dizziness

Study	Mirtazapine Events	Mirtazapine No events	Placebo Events	Placebo No events	Risk ratio with 95% CI	Weight (%)
Blier 2010	4	21	2	26	2.24 [0.45, 11.20]	18.22
Halikas 1995	11	39	4	46	2.75 [0.94, 8.06]	32.99
Kinoshita - 2009	15	195	1	69	5.00 [0.67, 37.17]	12.59
Organon UK unpublished	3	61	1	58	2.77 [0.30, 25.86]	10.41
Smith 1990	3	47	3	47	1.00 [0.21, 4.72]	19.33
Xiao 2021	4	64	0	68	9.00 [0.49, 164.00]	6.46
**Overall**					2.54 [1.18, 5.45]	

Heterogeneity: $\tau^2 = 0.16$, $I^2 = 17.29\%$, $H^2 = 1.21$

Test of $\theta_i = \theta_j$: Q(5) = 2.60, p = 0.76

Test of $\theta = 0$: z = 2.39, p = 0.02

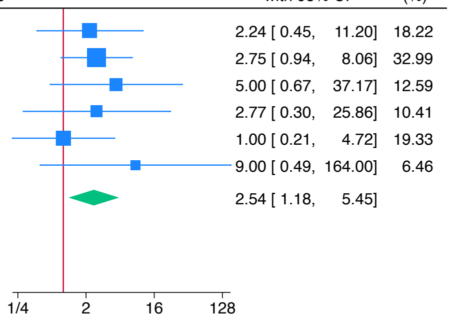

Random-effects Sidik–Jonkman model

Supplementary Figure 13: Meta-analysis of mirtazapine versus placebo on increased appetite

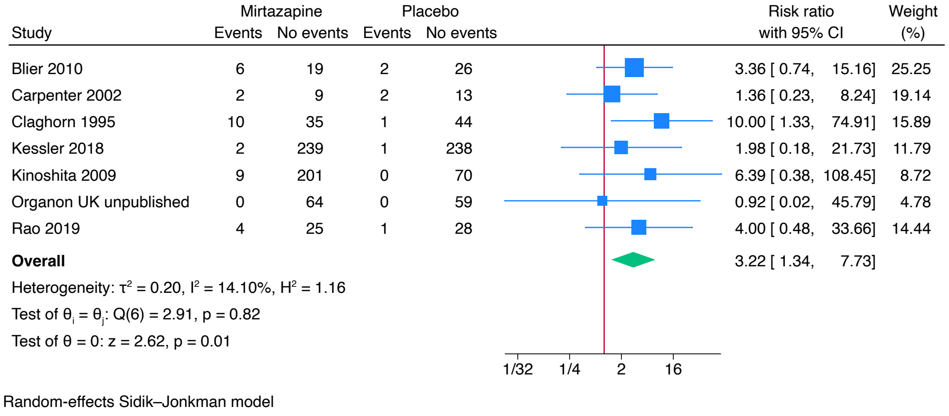

Supplementary Figure 14: Meta-analysis of mirtazapine versus placebo on headache

Study	Mirtazapine Events	Mirtazapine No events	Placebo Events	Placebo No events	Risk ratio with 95% CI	Weight (%)
Blier 2010	3	22	5	23	0.67 [0.18, 2.53]	9.58
Bremner 1995	2	48	3	47	0.67 [0.12, 3.82]	5.79
Carpenter 2002	0	11	3	12	0.19 [0.01, 3.35]	2.24
Halikas 1995	7	43	10	40	0.70 [0.29, 1.69]	18.98
Kessler 2018	4	237	4	235	0.99 [0.25, 3.92]	8.98
Kinoshita 2009	13	197	12	58	0.36 [0.17, 0.75]	24.90
Rao 2019	5	24	9	20	0.56 [0.21, 1.46]	16.49
Smith 1990	4	46	9	41	0.44 [0.15, 1.35]	13.04
**Overall**					0.54 [0.35, 0.83]	

Heterogeneity: $\tau^2 = 0.06$, $I^2 = 14.36\%$, $H^2 = 1.17$

Test of $\theta_i = \theta_j$: $Q(7) = 3.01$, $p = 0.88$

Test of $\theta = 0$: $z = -2.81$, $p = 0.00$

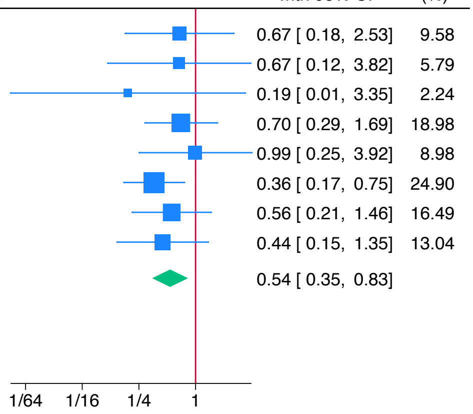

Random-effects Sidik–Jonkman model

Supplementary Figure 15: Meta-analysis of mirtazapine versus placebo on amblyopia

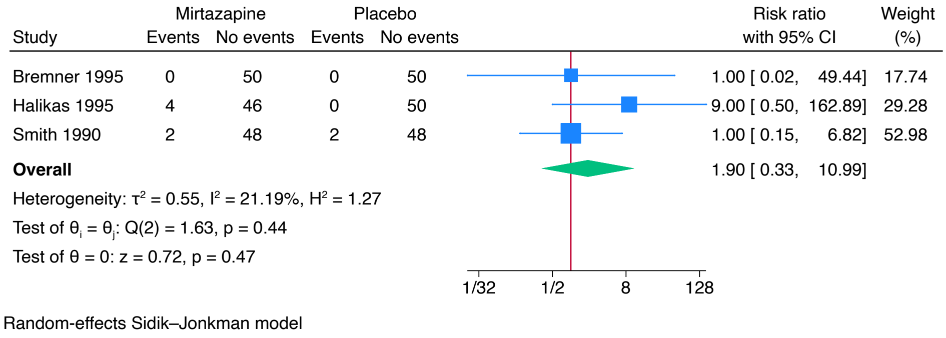

Supplementary Figure 16: Meta-analysis of mirtazapine versus placebo on asthenia

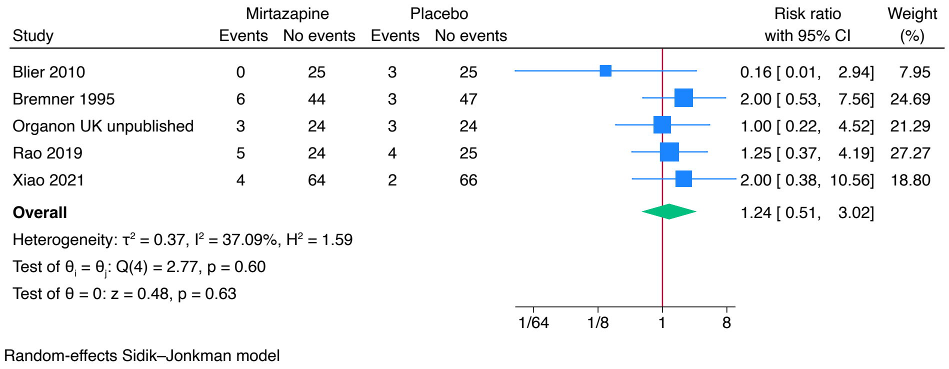

Supplementary Figure 17: Meta-analysis of mirtazapine versus placebo on blurred vision

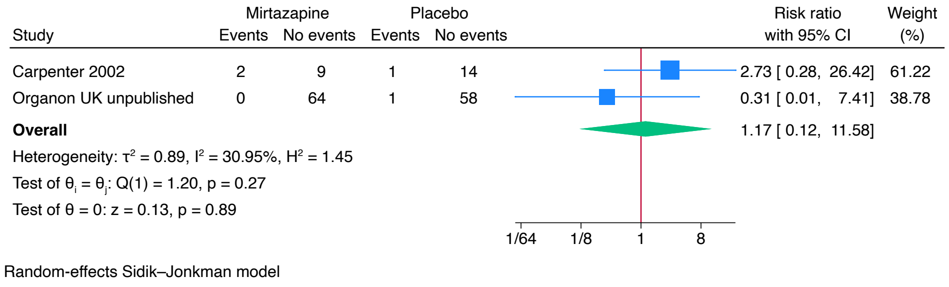

Supplementary Figure 18: Meta-analysis of mirtazapine versus placebo on constipation

Study	Mirtazapine Events	Mirtazapine No events	Placebo Events	Placebo No events	Risk ratio with 95% CI	Weight (%)
Blier 2010	0	25	3	25	0.16 [0.01, 2.94]	6.59
Bremner 1995	4	46	3	47	1.33 [0.31, 5.65]	14.63
Carpenter 2002	2	9	2	13	1.36 [0.23, 8.24]	12.00
Halikas 1995	9	41	8	42	1.12 [0.47, 2.68]	19.61
Kinoshita - 2009	27	183	3	67	3.00 [0.94, 9.59]	17.02
Organon UK unpublished	5	59	0	59	10.15 [0.57, 179.75]	6.73
Smith 1990	7	43	2	48	3.50 [0.76, 16.03]	14.02
Xiao 2021	1	67	3	65	0.33 [0.04, 3.13]	9.40
**Overall**					1.49 [0.62, 3.57]	

Heterogeneity: $\tau^2 = 0.82$, $I^2 = 57.18\%$, $H^2 = 2.34$

Test of $\theta_i = \theta_j$: Q(7) = 8.72, p = 0.27

Test of $\theta = 0$: z = 0.89, p = 0.38

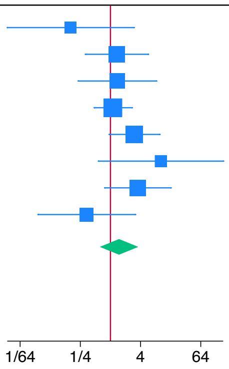

Random-effects Sidik–Jonkman model

Supplementary Figure 19: Meta-analysis of mirtazapine versus placebo on dyspepsia

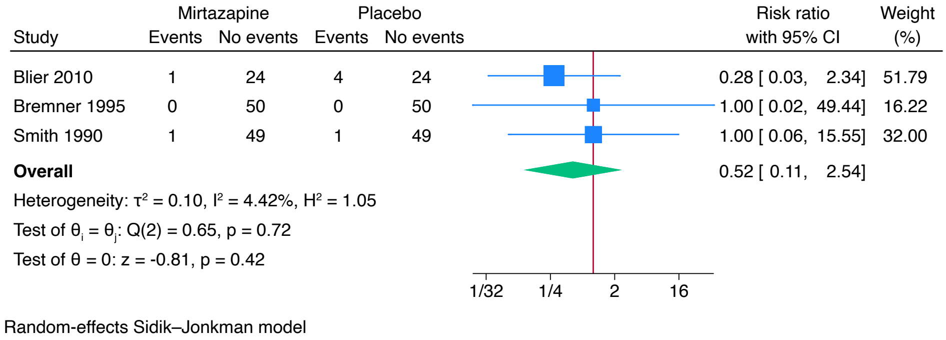

Supplementary Figure 20: Meta-analysis of mirtazapine versus placebo on insomnia

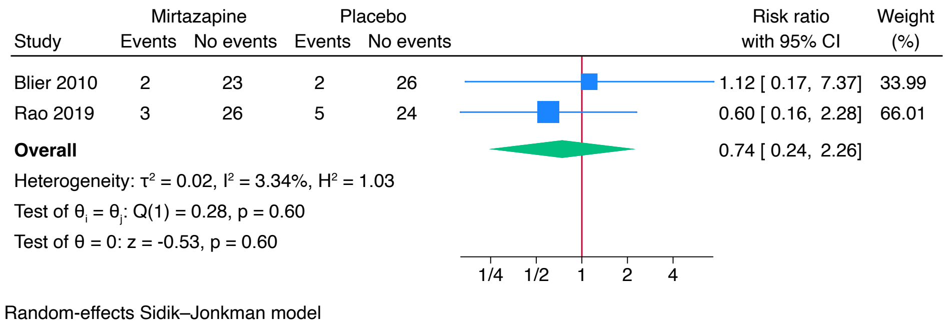

Supplementary Figure 21: Meta-analysis of mirtazapine versus placebo on nausea

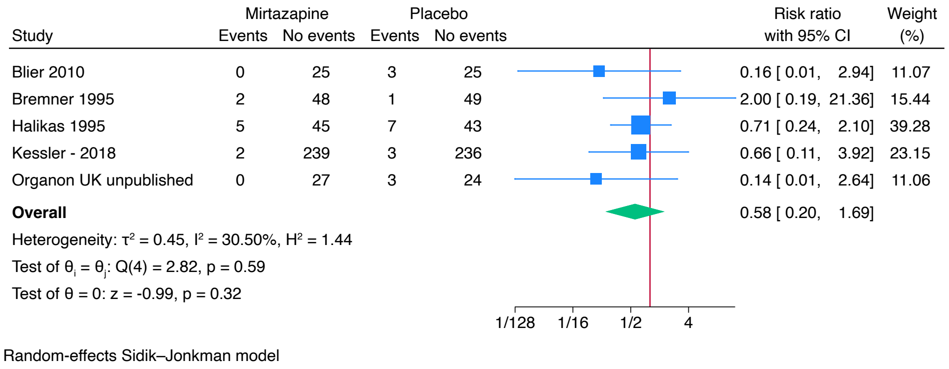

Supplementary Figure 22: Meta-analysis of mirtazapine versus placebo on sedation

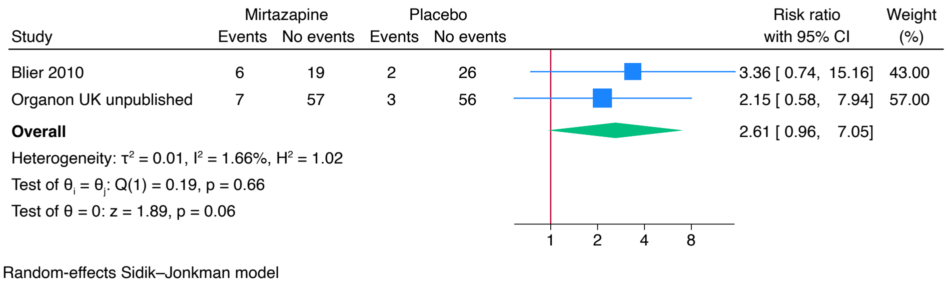

Supplementary Figure 23: Meta-analysis of mirtazapine versus placebo on stomach discomfort

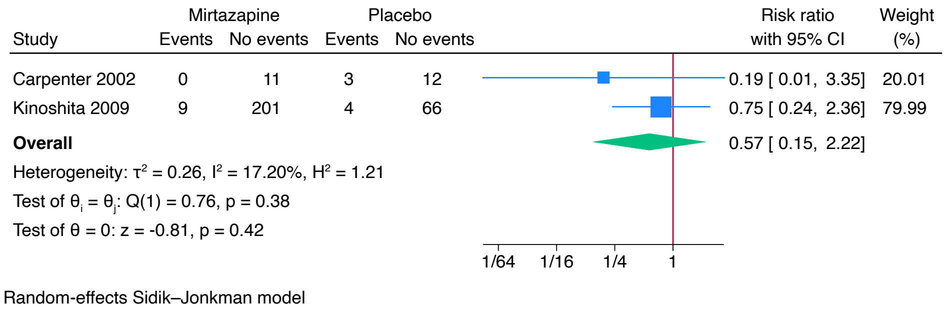

Supplementary Figure 24: Meta-analysis of mirtazapine versus placebo on tachycardia

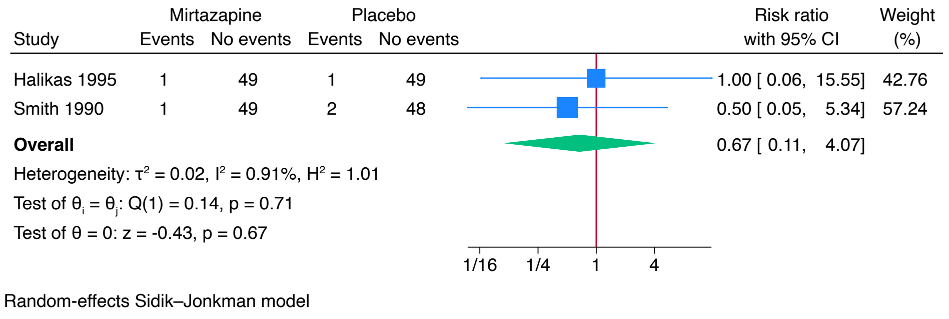

Supplementary Figure 25: Meta-analysis of mirtazapine versus placebo on tremor

Study	Mirtazapine Events	Mirtazapine No events	Placebo Events	Placebo No events	Risk ratio with 95% CI	Weight (%)
Blier 2010	0	25	2	26	0.22 [0.01, 4.44]	23.38
Bremner 1995	1	49	0	50	3.00 [0.13, 71.92]	21.02
Carpenter 2002	1	10	3	12	0.45 [0.05, 3.81]	41.10
Smith 1990	0	50	0	50	1.00 [0.02, 49.44]	14.51
**Overall**					0.64 [0.14, 3.02]	

Heterogeneity: $\tau^2 = 0.34$, $I^2 = 13.21\%$, $H^2 = 1.15$

Test of $\theta_i = \theta_j$: Q(3) = 1.53, p = 0.67

Test of $\theta = 0$: z = -0.56, p = 0.57

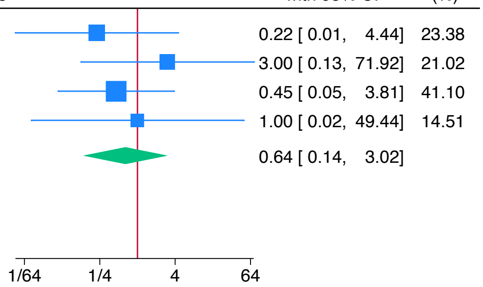

Random-effects Sidik–Jonkman model

Supplementary Figure 26: Meta-analysis of mirtazapine versus placebo on HDRS-17

Study	Mirtazapine N	Mirtazapine Mean	Mirtazapine SD	Placebo N	Placebo Mean	Placebo SD	Mean diff. with 95% CI	Weight (%)
Carpenter 2002	11	10.7	7	15	17.3	8.2	-6.60 [-12.61, -0.59]	4.41
Jacobson 1990	45	-10.24	5.94	48	-8.48	7.31	-1.76 [-4.48, 0.96]	14.86
Kinoshita 2009 (15 mg)	65	-13.3	6.8	24	-10.4	7.5	-2.90 [-6.17, 0.37]	11.66
Kinoshita 2009 (30 mg)	66	-13.8	6.9	23	-10.4	7.5	-3.40 [-6.75, -0.05]	11.30
Kinoshita 2009 (45 mg)	69	-11.9	7.6	23	-10.4	7.5	-1.50 [-5.07, 2.07]	10.29
Organon 3-020	38	-9.15	7.24	39	-4.46	5.7	-4.69 [-7.60, -1.78]	13.67
Organon UK unpublished	64	-11.78	7.19	61	-9.3	8.17	-2.48 [-5.17, 0.21]	15.01
Xiao 2021	59	7.09	5.42	56	8.38	6.59	-1.29 [-3.49, 0.91]	18.80
**Overall**							-2.69 [-4.03, -1.34]	

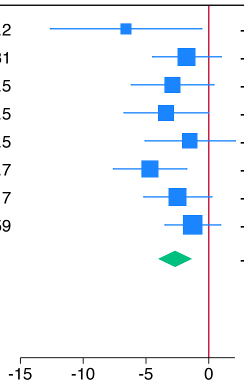

Heterogeneity: $\tau^2 = 1.24$, $I^2 = 33.82\%$, $H^2 = 1.51$

Test of $\theta_i = \theta_j$: $Q(7) = 6.04$, $p = 0.54$

Test of $\theta = 0$: $z = -3.92$, $p = 0.00$

Random-effects Sidik–Jonkman model

Supplementary Figure 27: Meta-analysis of mirtazapine versus placebo on MADRS, BDI, and HDRS-6

Study	Mirtazapine N	Mirtazapine Mean	Mirtazapine SD	Placebo N	Placebo Mean	Placebo SD	Hedges's g with 95% CI	Weight (%)
Jacobson 1990	45	-4.27	3.53	48	-3.83	4.59	-0.11 [-0.51, 0.30]	16.63
Kessler 2018	214	18	12.3	217	19.7	12.4	-0.14 [-0.33, 0.05]	36.20
Organon 3-020	38	-4.24	3.85	39	-2.13	2.93	-0.61 [-1.06, -0.16]	14.11
Organon UK unpublished	64	-5.39	3.88	61	-4.16	3.78	-0.32 [-0.67, 0.03]	20.01
Vartiainen 1994	37	15.3	11.2	30	17.2	10.6	-0.17 [-0.65, 0.31]	13.05
**Overall**							-0.24 [-0.44, -0.04]	

Heterogeneity: $\tau^2 = 0.02$, $I^2 = 37.80\%$, $H^2 = 1.61$

Test of $\theta_i = \theta_j$: Q(4) = 4.24, p = 0.37

Test of $\theta = 0$: z = -2.38, p = 0.02

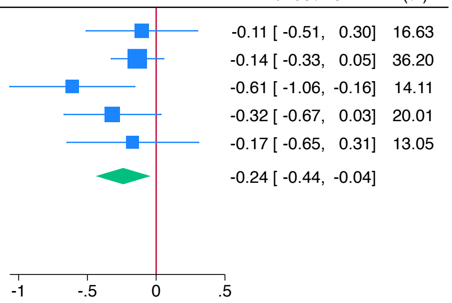

Random-effects Sidik–Jonkman model

Supplementary Figure 28: Meta-analysis of mirtazapine versus placebo on suicides or suicide attempts (sensitivity analysis)

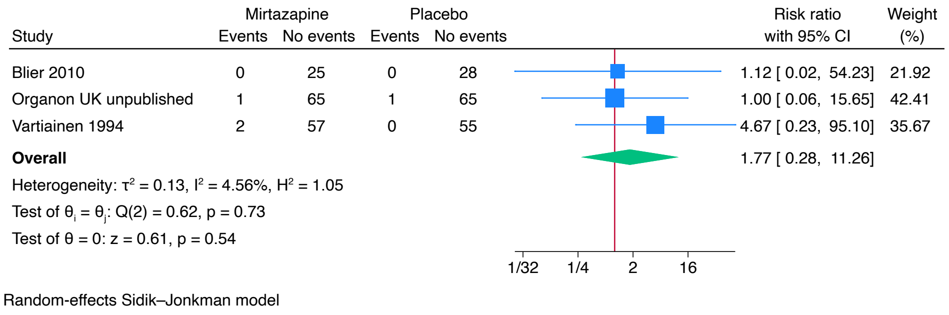

Supplementary Figure 29: Meta-analysis of mirtazapine versus placebo on serious adverse events (sensitivity analysis)

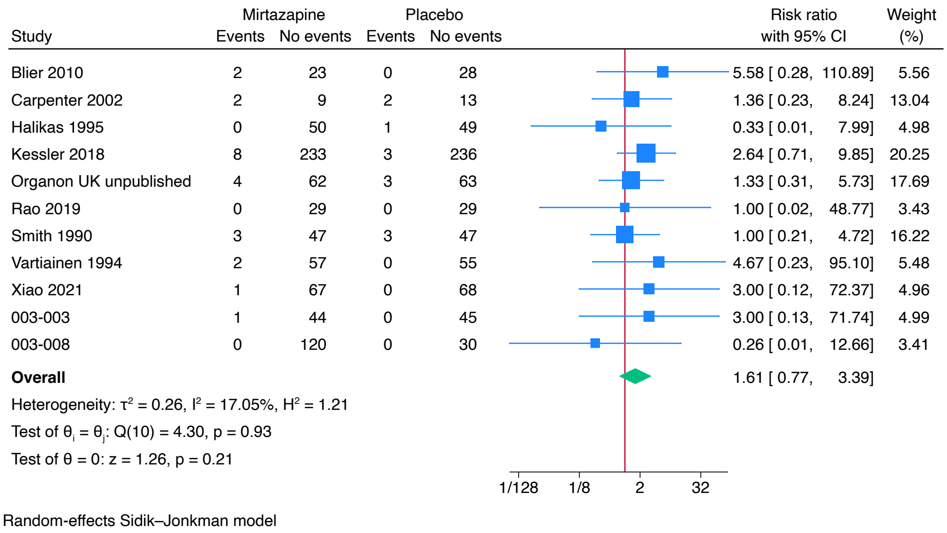

Supplementary Figure 30: Meta-analysis of mirtazapine versus placebo on sexual dysfunction (sensitivity analysis)

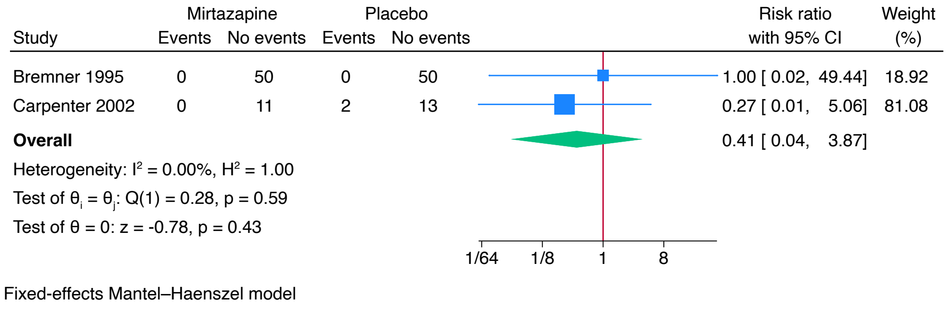

Supplementary Figure 31: Meta-analysis of mirtazapine versus placebo on anxiety (sensitivity analysis)

Study	Mirtazapine Events	Mirtazapine No events	Placebo Events	Placebo No events	Risk ratio with 95% CI	Weight (%)
Blier 2010	2	23	0	28	5.58 [0.28, 110.89]	48.60
Xiao 2021	0	68	0	68	1.00 [0.02, 49.68]	51.40
**Overall**					3.22 [0.35, 29.51]	

Heterogeneity: $I^2 = 0.00\%$, $H^2 = 1.00$

Test of $\theta_i = \theta_j$: Q(1) = 0.47, p = 0.49

Test of $\theta = 0$: z = 1.04, p = 0.30

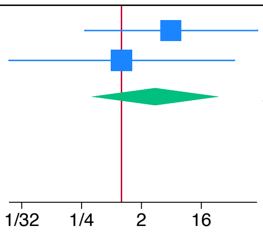

Fixed-effects Mantel–Haenszel model

Supplementary Figure 32: Meta-analysis of mirtazapine versus placebo on non-serious adverse events (sensitivity analysis)

	Mirtazapine		Placebo		Risk ratio	Weight
Study	Events	No events	Events	No events	with 95% CI	(%)
Blier 2010	12	13	9	19	1.49 [0.76, 2.93]	7.61
Bremner 1995	27	23	15	35	1.80 [1.10, 2.95]	13.45
Carpenter 2002	3	8	3	12	1.36 [0.34, 5.52]	2.28
Claghorn 1995	28	17	2	43	14.00 [3.54, 55.30]	1.79
Halikas 1995	27	23	11	39	2.45 [1.37, 4.39]	9.86
Kessler 2018	41	200	5	234	8.13 [3.27, 20.22]	4.50
Kinoshita 2009 (15 mg)	100	110	21	49	1.59 [1.08, 2.33]	28.24
Organon UK unpublished	15	12	13	14	1.15 [0.69, 1.93]	11.65
Smith 1990	34	16	8	42	4.25 [2.19, 8.25]	7.17
Xiao 2021	28	40	15	53	1.87 [1.10, 3.17]	13.45
**Overall**					2.38 [1.97, 2.88]	

Heterogeneity: $I^2 = 72.45\%$, $H^2 = 3.63$

Test of $\theta_i = \theta_j$: $Q(9) = 32.66$, $p = 0.00$

Test of $\theta = 0$: $z = 8.99$, $p = 0.00$

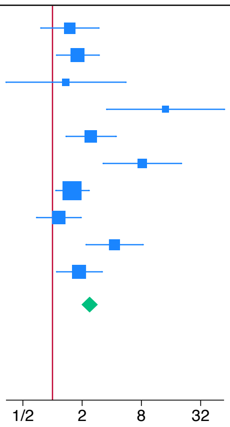

Fixed-effects Mantel–Haenszel model

Supplementary Figure 33: Meta-analysis of mirtazapine versus placebo on somnolence (sensitivity analysis)

Study	Mirtazapine Events	Mirtazapine No events	Placebo Events	Placebo No events	Risk ratio with 95% CI	Weight (%)
Blier 2010	2	23	3	25	0.75 [0.14, 4.11]	3.64
Bremner 1995	23	27	11	39	2.09 [1.15, 3.82]	14.16
Carpenter 2002	1	10	2	13	0.68 [0.07, 6.61]	2.18
Claghorn 1995	28	17	2	43	14.00 [3.54, 55.30]	2.57
Halikas 1995	27	23	11	39	2.45 [1.37, 4.39]	14.16
Kessler - 2018	41	200	5	234	8.13 [3.27, 20.22]	6.46
Kinoshita - 2009	100	110	21	49	1.59 [1.08, 2.33]	40.54
Organon UK unpublished	2	62	4	55	0.46 [0.09, 2.42]	5.36
Smith 1990	34	16	8	42	4.25 [2.19, 8.25]	10.30
Xiao 2021	6	62	0	68	13.00 [0.75, 226.32]	0.64
**Overall**					2.76 [2.19, 3.48]	

Heterogeneity: $I^2 = 70.61\%$, $H^2 = 3.40$

Test of $\theta_i = \theta_j$: $Q(9) = 30.62$, $p = 0.00$

Test of $\theta = 0$: $z = 8.58$, $p = 0.00$

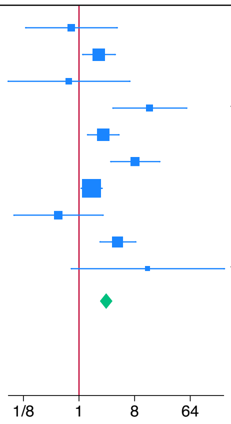

Fixed-effects Mantel–Haenszel model

Supplementary Figure 34: Meta-analysis of mirtazapine versus placebo on weight gain (sensitivity analysis)

Study	Mirtazapine Events	Mirtazapine No events	Placebo Events	Placebo No events	Risk ratio with 95% CI	Weight (%)
Blier 2010	3	22	0	28	7.81 [0.42, 144.12]	3.56
Carpenter 2002	3	8	3	12	1.36 [0.34, 5.52]	19.10
Claghorn 1995	9	36	0	45	19.00 [1.14, 316.95]	3.76
Kessler 2018	24	217	7	232	3.40 [1.49, 7.74]	52.90
Kinoshita 2009	15	195	0	70	10.43 [0.63, 172.10]	5.63
Rao 2019	5	24	1	28	5.00 [0.62, 40.20]	7.53
Smith 1990	16	34	1	49	16.00 [2.20, 116.10]	7.53
**Overall**					5.22 [2.91, 9.35]	

Heterogeneity: $I^2 = 13.39\%$, $H^2 = 1.15$

Test of $\theta_i = \theta_j$: $Q(6) = 6.93$, $p = 0.33$

Test of $\theta = 0$: $z = 5.55$, $p = 0.00$

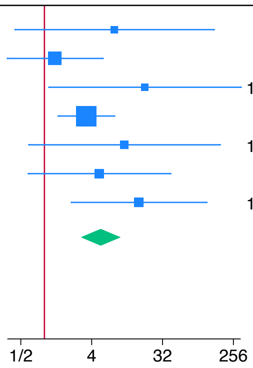

Fixed-effects Mantel–Haenszel model

Supplementary Figure 35: Meta-analysis of mirtazapine versus placebo on dry mouth (sensitivity analysis)

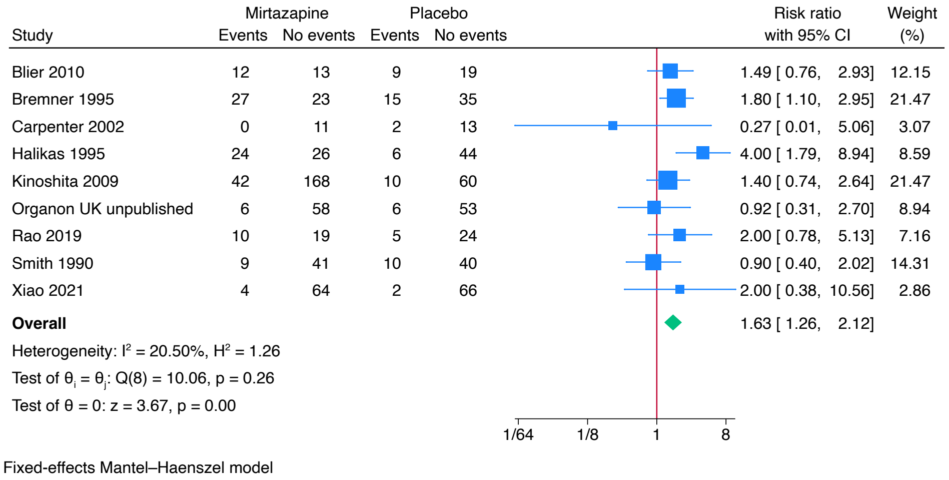

Supplementary Figure 36: Meta-analysis of mirtazapine versus placebo on dizziness (sensitivity analysis)

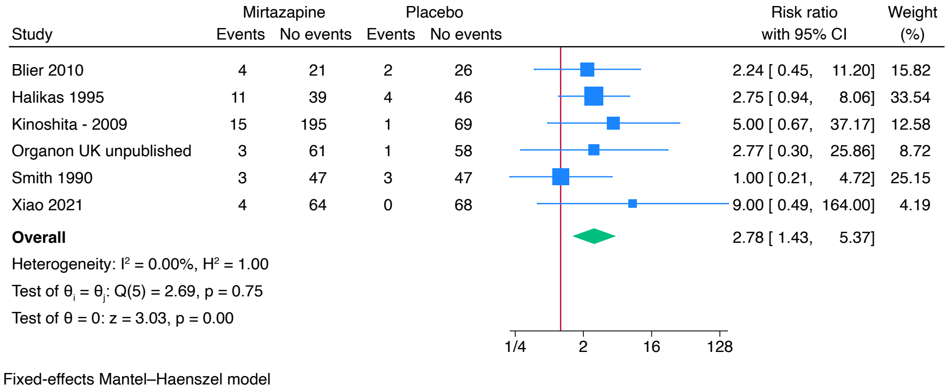

Supplementary Figure 37: Meta-analysis of mirtazapine versus placebo on increased appetite (sensitivity analysis)

Study	Mirtazapine Events	Mirtazapine No events	Placebo Events	Placebo No events	Risk ratio with 95% CI	Weight (%)
Blier 2010	6	19	2	26	3.36 [0.74, 15.16]	24.03
Carpenter 2002	2	9	2	13	1.36 [0.23, 8.24]	21.55
Claghorn 1995	10	35	1	44	10.00 [1.33, 74.91]	12.74
Kessler 2018	2	239	1	238	1.98 [0.18, 21.73]	12.79
Kinoshita 2009	9	201	0	70	6.39 [0.38, 108.45]	9.53
Organon UK unpublished	0	64	0	59	0.92 [0.02, 45.79]	6.62
Rao 2019	4	25	1	28	4.00 [0.48, 33.66]	12.74
**Overall**					3.81 [1.75, 8.30]	

Heterogeneity: $I^2 = 0.00\%$, $H^2 = 1.00$

Test of $\theta_i = \theta_j$: Q(6) = 3.08, p = 0.80

Test of $\theta = 0$: z = 3.36, p = 0.00

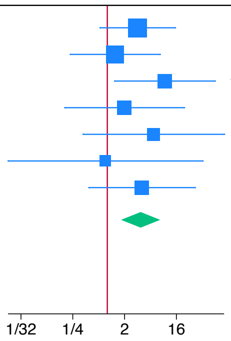

Fixed-effects Mantel–Haenszel model

Supplementary Figure 38: Meta-analysis of mirtazapine versus placebo on headache (sensitivity analysis)

Study	Mirtazapine Events	Mirtazapine No events	Placebo Events	Placebo No events	Risk ratio with 95% CI	Weight (%)
Blier 2010	3	22	5	23	0.67 [0.18, 2.53]	7.77
Bremner 1995	2	48	3	47	0.67 [0.12, 3.82]	4.94
Carpenter 2002	0	11	3	12	0.19 [0.01, 3.35]	4.94
Halikas 1995	7	43	10	40	0.70 [0.29, 1.69]	16.47
Kessler 2018	4	237	4	235	0.99 [0.25, 3.92]	6.61
Kinoshita 2009	13	197	12	58	0.36 [0.17, 0.75]	29.64
Rao 2019	5	24	9	20	0.56 [0.21, 1.46]	14.82
Smith 1990	4	46	9	41	0.44 [0.15, 1.35]	14.82
**Overall**					0.53 [0.36, 0.78]	

Heterogeneity: $I^2 = 0.00\%$, $H^2 = 1.00$

Test of $\theta_i = \theta_j$: Q(7) = 3.01, p = 0.88

Test of θ = 0: z = -3.21, p = 0.00

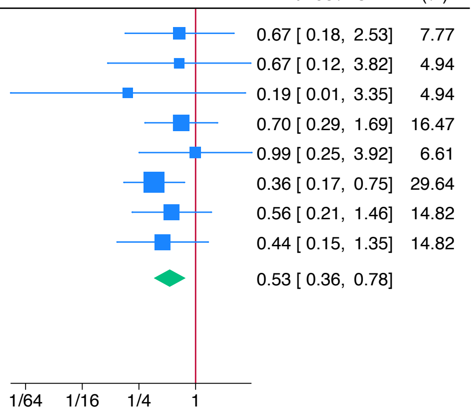

Fixed-effects Mantel–Haenszel model

Supplementary Figure 39: Meta-analysis of mirtazapine versus placebo on amblyopia (sensitivity analysis)

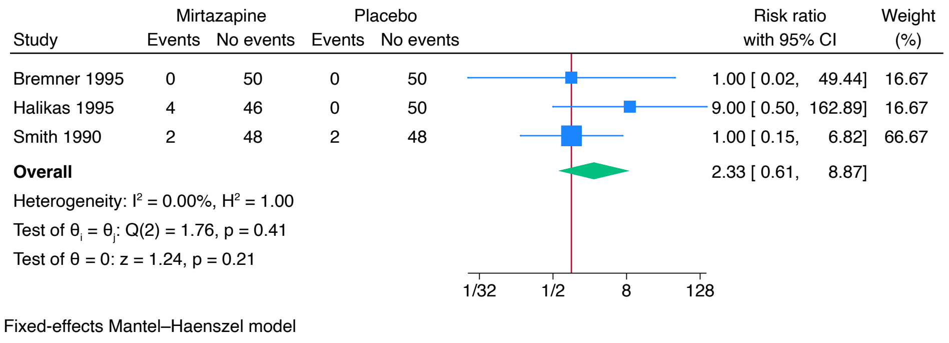

Supplementary Figure 40: Meta-analysis of mirtazapine versus placebo on asthenia (sensitivity analysis)

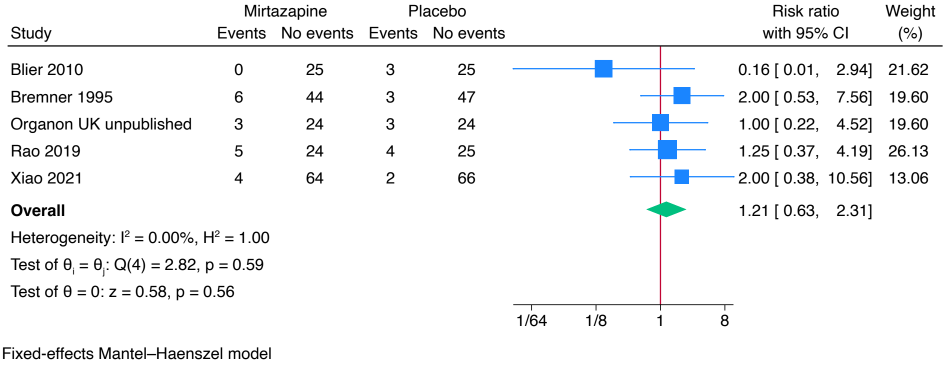

Supplementary Figure 41: Meta-analysis of mirtazapine versus placebo on blurred vision (sensitivity analysis)

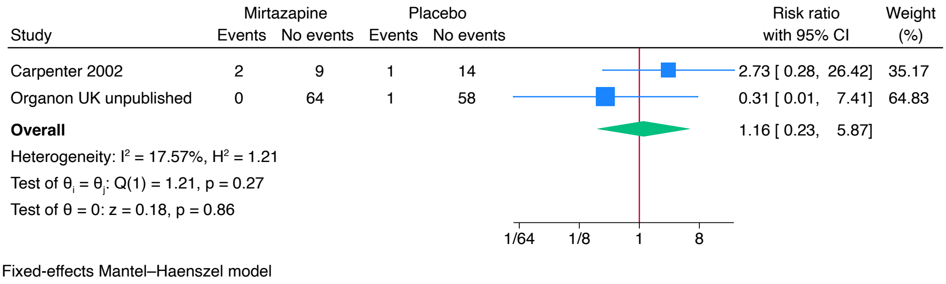

STATA

Supplementary Figure 42: Meta-analysis of mirtazapine versus placebo on constipation (sensitivity analysis)

Study	Mirtazapine Events	Mirtazapine No events	Placebo Events	Placebo No events	Risk ratio with 95% CI	Weight (%)
Blier 2010	0	25	3	25	0.16 [0.01, 2.94]	12.72
Bremner 1995	4	46	3	47	1.33 [0.31, 5.65]	11.53
Carpenter 2002	2	9	2	13	1.36 [0.23, 8.24]	6.50
Halikas 1995	9	41	8	42	1.12 [0.47, 2.68]	30.74
Kinoshita - 2009	27	183	3	67	3.00 [0.94, 9.59]	17.29
Organon UK unpublished	5	59	0	59	10.15 [0.57, 179.75]	2.00
Smith 1990	7	43	2	48	3.50 [0.76, 16.03]	7.69
Xiao 2021	1	67	3	65	0.33 [0.04, 3.13]	11.53
**Overall**					1.64 [1.01, 2.66]	

Heterogeneity: $I^2 = 20.29\%$, $H^2 = 1.25$

Test of $\theta_i = \theta_j$: Q(7) = 8.78, p = 0.27

Test of $\theta = 0$: z = 2.00, p = 0.05

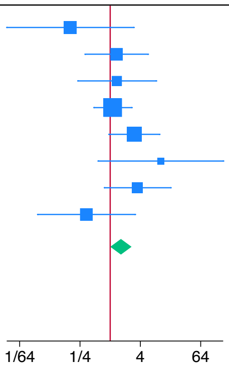

Fixed-effects Mantel–Haenszel model

Supplementary Figure 43: Meta-analysis of mirtazapine versus placebo on dyspepsia (sensitivity analysis)

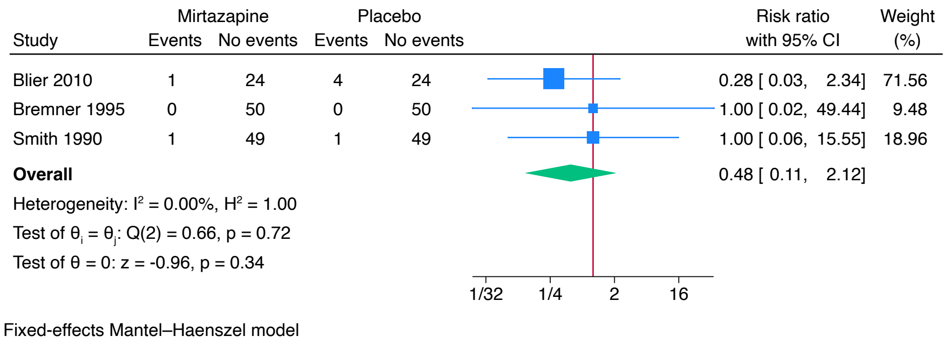

Supplementary Figure 44: Meta-analysis of mirtazapine versus placebo on insomnia (sensitivity analysis)

Study	Mirtazapine Events	Mirtazapine No events	Placebo Events	Placebo No events	Risk ratio with 95% CI	Weight (%)
Blier 2010	2	23	2	26	1.12 [0.17, 7.37]	27.40
Rao 2019	3	26	5	24	0.60 [0.16, 2.28]	72.60
**Overall**					0.74 [0.25, 2.18]	

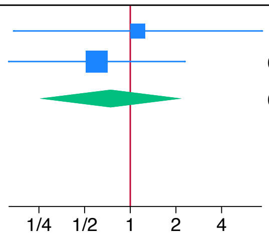

Heterogeneity: $I^2 = 0.00\%$, $H^2 = 1.00$

Test of $\theta_i = \theta_j$: Q(1) = 0.28, p = 0.60

Test of $\theta = 0$: z = -0.54, p = 0.59

Fixed-effects Mantel–Haenszel model

Supplementary Figure 45: Meta-analysis of mirtazapine versus placebo on nausea (sensitivity analysis)

Study	Mirtazapine Events	Mirtazapine No events	Placebo Events	Placebo No events	Risk ratio with 95% CI	Weight (%)
Blier 2010	0	25	3	25	0.16 [0.01, 2.94]	18.57
Bremner 1995	2	48	1	49	2.00 [0.19, 21.36]	5.61
Halikas 1995	5	45	7	43	0.71 [0.24, 2.10]	39.28
Kessler - 2018	2	239	3	236	0.66 [0.11, 3.92]	16.90
Organon UK unpublished	0	27	3	24	0.14 [0.01, 2.64]	19.64
**Overall**					0.56 [0.27, 1.19]	

Heterogeneity: $I^2 = 0.00\%$, $H^2 = 1.00$

Test of $\theta_i = \theta_j$: $Q(4) = 2.89$, $p = 0.58$

Test of $\theta = 0$: $z = -1.50$, $p = 0.13$

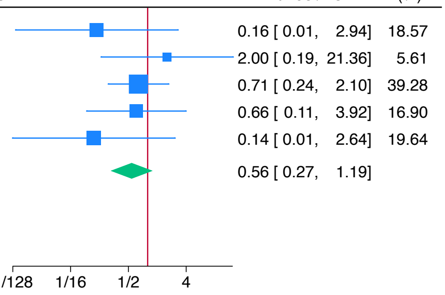

Fixed-effects Mantel–Haenszel model

Supplementary Figure 46: Meta-analysis of mirtazapine versus placebo on sedation (sensitivity analysis)

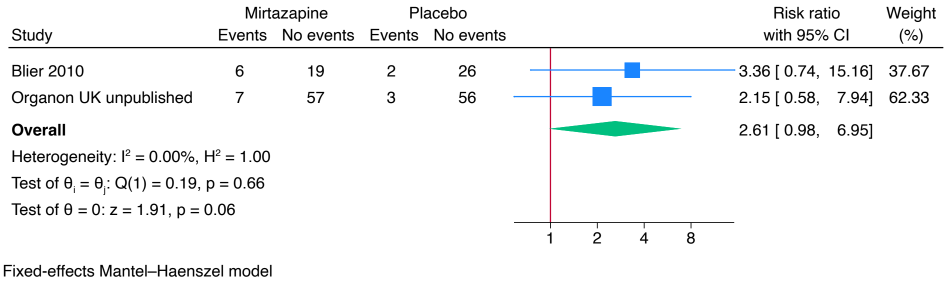

Supplementary Figure 47: Meta-analysis of mirtazapine versus placebo on stomach discomfort (sensitivity analysis)

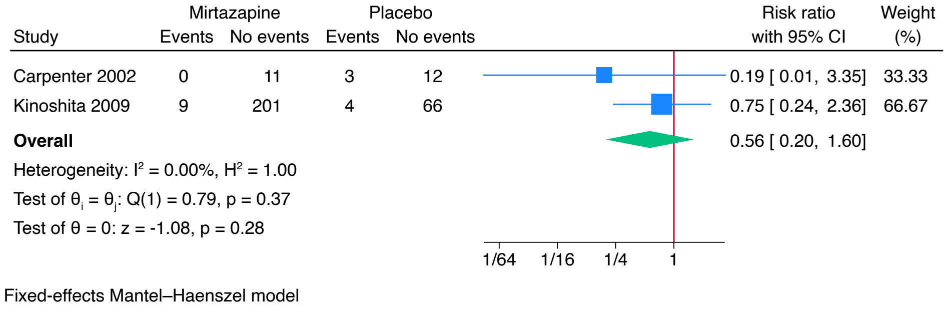

Supplementary Figure 48: Meta-analysis of mirtazapine versus placebo on tachycardia (sensitivity analysis)

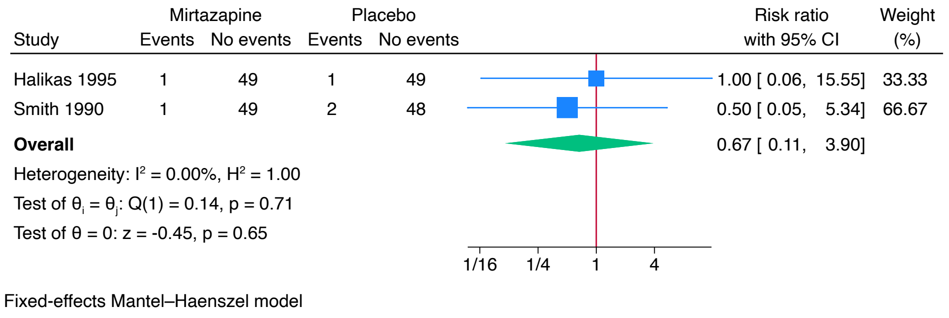

Supplementary Figure 49: Meta-analysis of mirtazapine versus placebo on tremor (sensitivity analysis)

Study	Mirtazapine Events	Mirtazapine No events	Placebo Events	Placebo No events	Risk ratio with 95% CI	Weight (%)
Blier 2010	0	25	2	26	0.22 [0.01, 4.44]	40.05
Bremner 1995	1	49	0	50	3.00 [0.13, 71.92]	8.47
Carpenter 2002	1	10	3	12	0.45 [0.05, 3.81]	43.01
Smith 1990	0	50	0	50	1.00 [0.02, 49.44]	8.47
**Overall**					0.62 [0.17, 2.25]	

Heterogeneity: $I^2 = 0.00\%$, $H^2 = 1.00$

Test of $\theta_i = \theta_j$: Q(3) = 1.53, p = 0.67

Test of $\theta = 0$: z = -0.72, p = 0.47

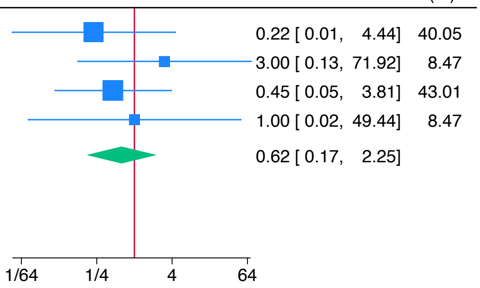

Fixed-effects Mantel–Haenszel model

Supplementary Figure 50: Meta-analysis of mirtazapine versus placebo on HDRS-17 (sensitivity analysis)

Study	Mirtazapine N	Mirtazapine Mean	Mirtazapine SD	Placebo N	Placebo Mean	Placebo SD	Mean diff. with 95% CI	Weight (%)
Carpenter 2002	11	10.7	7	15	17.3	8.2	-6.60 [-12.61, -0.59]	3.12
Jacobson 1990	45	-10.24	5.94	48	-8.48	7.31	-1.76 [-4.48, 0.96]	15.27
Kinoshita 2009 (15 mg)	65	-13.3	6.8	24	-10.4	7.5	-2.90 [-6.17, 0.37]	10.53
Kinoshita 2009 (30 mg)	66	-13.8	6.9	23	-10.4	7.5	-3.40 [-6.75, -0.05]	10.06
Kinoshita 2009 (45 mg)	69	-11.9	7.6	23	-10.4	7.5	-1.50 [-5.07, 2.07]	8.83
Organon 3-020	38	-9.15	7.24	39	-4.46	5.7	-4.69 [-7.60, -1.78]	13.36
Organon UK unpublished	64	-11.78	7.19	61	-9.3	8.17	-2.48 [-5.17, 0.21]	15.54
Xiao 2021	59	7.09	5.42	56	8.38	6.59	-1.29 [-3.49, 0.91]	23.30
**Overall**							-2.57 [-3.63, -1.50]	

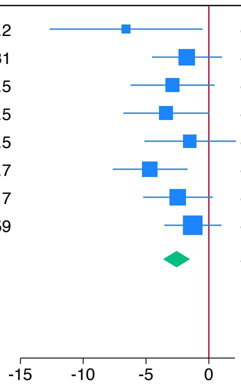

Heterogeneity: $I^2 = 0.00\%$, $H^2 = 1.00$

Test of $\theta_i = \theta_j$: $Q(7) = 6.04$, $p = 0.54$

Test of $\theta = 0$: $z = -4.74$, $p = 0.00$

Fixed-effects inverse-variance model

Supplementary Figure 51: Meta-analysis of mirtazapine versus placebo on MADRS, BDI, and HDRS-6 (sensitivity analysis)

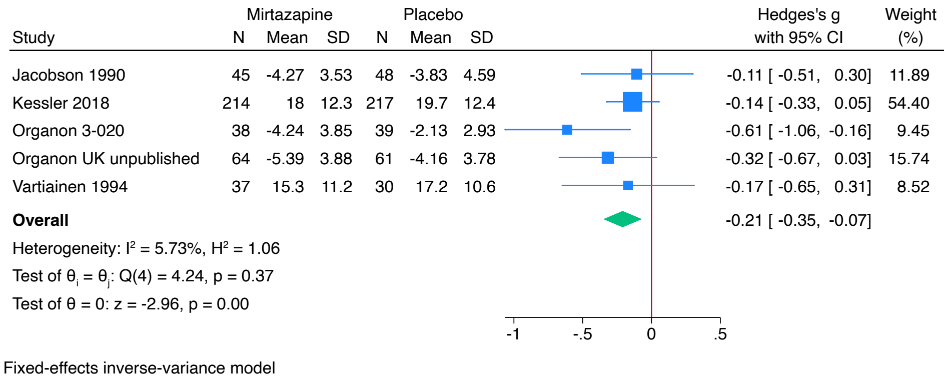

PRISMA 2020 Checklist

Section and Topic	Item #	Checklist item	Location where item is reported
**TITLE**			
Title	1	Identify the report as a systematic review.	Title
**ABSTRACT**			
Abstract	2	See the PRISMA 2020 for Abstracts checklist.	Abstract
**INTRODUCTION**			
Rationale	3	Describe the rationale for the review in the context of existing knowledge.	Introduction
Objectives	4	Provide an explicit statement of the objective(s) or question(s) the review addresses.	Introduction
**METHODS**			
Eligibility criteria	5	Specify the inclusion and exclusion criteria for the review and how studies were grouped for the syntheses.	Selection criteria
Information sources	6	Specify all databases, registers, websites, organisations, reference lists and other sources searched or consulted to identify studies. Specify the date when each source was last searched or consulted.	Search strategy
Search strategy	7	Present the full search strategies for all databases, registers and websites, including any filters and limits used.	Search strategy & Supplementary
Selection process	8	Specify the methods used to decide whether a study met the inclusion criteria of the review, including how many reviewers screened each record and each report retrieved, whether they worked independently, and if applicable, details of automation tools used in the process.	Data extraction and risk of bias assessment
Data collection process	9	Specify the methods used to collect data from reports, including how many reviewers collected data from each report, whether they worked independently, any processes for obtaining or confirming data from study investigators, and if applicable, details of automation tools used in the process.	Data extraction and risk of bias assessment
Data items	10a	List and define all outcomes for which data were sought. Specify whether all results that were compatible with each outcome domain in each study were sought (e.g. for all measures, time points, analyses), and if not, the methods used to decide which results to collect.	Outcomes and Subgroup analysis
	10b	List and define all other variables for which data were sought (e.g. participant and intervention characteristics, funding sources). Describe any assumptions made about any missing or unclear information.	Protocol
Study risk of bias assessment	11	Specify the methods used to assess risk of bias in the included studies, including details of the tool(s) used, how many reviewers assessed each study and whether they worked independently, and if applicable, details of automation tools used in the process.	Data extraction and risk of bias assessment
Effect measures	12	Specify for each outcome the effect measure(s) (e.g. risk ratio, mean difference) used in the synthesis or presentation of results.	Protocol
Synthesis methods	13a	Describe the processes used to decide which studies were eligible for each synthesis (e.g. tabulating the study intervention characteristics and comparing against the planned groups for each synthesis (item #5)).	Protocol
	13b	Describe any methods required to prepare the data for presentation or synthesis, such as handling of missing summary statistics, or data conversions.	Protocol
	13c	Describe any methods used to tabulate or visually display results of individual studies and syntheses.	Assessment of statistical and clinical significance

PRISMA 2020 Checklist

Section and Topic	Item #	Checklist item	Location where item is reported
	13d	Describe any methods used to synthesize results and provide a rationale for the choice(s). If meta-analysis was performed, describe the model(s), method(s) to identify the presence and extent of statistical heterogeneity, and software package(s) used.	Assessment of statistical and clinical significance
	13e	Describe any methods used to explore possible causes of heterogeneity among study results (e.g. subgroup analysis, meta-regression).	Protocol
	13f	Describe any sensitivity analyses conducted to assess robustness of the synthesized results.	Protocol & Results
Reporting bias assessment	14	Describe any methods used to assess risk of bias due to missing results in a synthesis (arising from reporting biases).	Protocol
Certainty assessment	15	Describe any methods used to assess certainty (or confidence) in the body of evidence for an outcome.	Protocol
**RESULTS**			
Study selection	16a	Describe the results of the search and selection process, from the number of records identified in the search to the number of studies included in the review, ideally using a flow diagram.	Figure 1
	16b	Cite studies that might appear to meet the inclusion criteria, but which were excluded, and explain why they were excluded.	Results
Study characteristics	17	Cite each included study and present its characteristics.	Table 1
Risk of bias in studies	18	Present assessments of risk of bias for each included study.	Results
Results of individual studies	19	For all outcomes, present, for each study: (a) summary statistics for each group (where appropriate) and (b) an effect estimate and its precision (e.g. confidence/credible interval), ideally using structured tables or plots.	Results
Results of syntheses	20a	For each synthesis, briefly summarise the characteristics and risk of bias among contributing studies.	Results
	20b	Present results of all statistical syntheses conducted. If meta-analysis was done, present for each the summary estimate and its precision (e.g. confidence/credible interval) and measures of statistical heterogeneity. If comparing groups, describe the direction of the effect.	Results
	20c	Present results of all investigations of possible causes of heterogeneity among study results.	Results
	20d	Present results of all sensitivity analyses conducted to assess the robustness of the synthesized results.	Results & Supplementary
Reporting biases	21	Present assessments of risk of bias due to missing results (arising from reporting biases) for each synthesis assessed.	Results
Certainty of evidence	22	Present assessments of certainty (or confidence) in the body of evidence for each outcome assessed.	Results
**DISCUSSION**			
Discussion	23a	Provide a general interpretation of the results in the context of other evidence.	Discussion
	23b	Discuss any limitations of the evidence included in the review.	Discussion
	23c	Discuss any limitations of the review processes used.	Discussion
	23d	Discuss implications of the results for practice, policy, and future research.	Discussion
**OTHER INFORMATION**			
Registration and	24a	Provide registration information for the review, including register name and registration number, or state that the review was not registered.	Methods

PRISMA 2020 Checklist

Section and Topic	Item #	Checklist item	Location where item is reported
protocol	24b	Indicate where the review protocol can be accessed, or state that a protocol was not prepared.	Methods
	24c	Describe and explain any amendments to information provided at registration or in the protocol.	Differences between the protocol and the review
Support	25	Describe sources of financial or non-financial support for the review, and the role of the funders or sponsors in the review.	Financial Support & Competing Interests
Competing interests	26	Declare any competing interests of review authors.	Competing Interests
Availability of data, code and other materials	27	Report which of the following are publicly available and where they can be found: template data collection forms; data extracted from included studies; data used for all analyses; analytic code; any other materials used in the review.	Data used for all analyses are available in the results.

From: Page MJ, McKenzie JE, Bossuyt PM, Boutron I, Hoffmann TC, Mulrow CD, et al. The PRISMA 2020 statement: an updated guideline for reporting systematic reviews. BMJ 2021;372:n71. doi: 10.1136/bmj.n71

For more information, visit: http://www.prisma-statement.org/

Search strategies for
‘Venlafaxine or Mirtazapine for major depressive disorder’
(C Kamp)
Updated searches performed 7 March 2024

**Total number of records identified:**	**10691 records**
**Number of duplicates excluded:**	**2770 records**
**Number of records in final list:**	**7921 records**
**Number of new records sent to authors:**	**763 records**

Cochrane Central Register of Controlled Trials (2024, Issue 2) in the Cochrane Library (813 hits)
#1 MeSH descriptor: [Venlafaxine Hydrochloride] explode all trees
#2 MeSH descriptor: [Mirtazapine] explode all trees
#3 (venlafaxin* or ef*exor* or mirtazapin* or org*3770 or remeron*)
#4 #1 or #2 #3
#5 MeSH descriptor: [Depressive Disorder, Major] explode all trees
#6 MeSH descriptor: [Depressive Disorder] this term only
#7 MeSH descriptor: [Seasonal Affective Disorder] explode all trees
#8 MeSH descriptor: [Dysthymic Disorder] explode all trees
#9 MeSH descriptor: [Depression] explode all trees
#10 MeSH descriptor: [Affective Symptoms] this term only
#11 ((depress* or affective or dysthym*) and (disorder* or disease* or symptom*))
#12 #5 or #6 or #7 or #8 or #9 or #10 or #11
#13 #4 and #12

MEDLINE Ovid (1946 to 7 March 2024) (2963 hits)
1. exp Venlafaxine Hydrochloride/
2. exp Mirtazapine/
3. (venlafaxin* or ef*exor* or mirtazapin* or org*3770 or remeron*).mp. [mp=title, abstract, original title, name of substance word, subject heading word, floating sub-heading word, keyword heading word, organism supplementary concept word, protocol supplementary concept word, rare disease supplementary concept word, unique identifier, synonyms]
4. 1 or 2 or 3
5. exp Depressive Disorder, Major/
6. Depressive Disorder/
7. exp Seasonal Affective Disorder/
8. exp Dysthymic Disorder/
9. exp Depression/
10. Affective Symptoms/
11. ((depress* or affective or dysthym*) and (disorder* or disease* or symptom*)).mp. [mp=title, abstract, original title, name of substance word, subject heading word, floating sub-heading word, keyword heading word, organism supplementary concept word, protocol supplementary concept word, rare disease supplementary concept word, unique identifier, synonyms]
12. 5 or 6 or 7 or 8 or 9 or 10 or 11
13. 4 and 12
14. (randomized controlled trial or controlled clinical trial).pt. or clinical trials as topic.sh. or trial.ti.
15. (random* or blind* or placebo* or meta-analys*).mp. [mp=title, abstract, original title, name of substance word, subject heading word, floating sub-heading word, keyword heading word, organism supplementary concept word, protocol supplementary concept word, rare disease supplementary concept word, unique identifier, synonyms]
16. 13 and (14 or 15)
17. limit 16 to ("adolescent (13 to 18 years)" or "young adult (19 to 24 years)" or "adult (19 to 44 years)" or "young adult and adult (19-24 and 19-44)" or "middle age (45 to 64 years)" or "middle aged (45 plus years)" or "all aged (65 and over)" or "aged (80 and over)")

Embase Ovid (1974 to 7 March 2024) (5650 hits)

1. exp venlafaxine/
2. exp mirtazapine/
3. (venlafaxin* or ef*exor* or mirtazapin* or org*3770 or remeron*).mp. [mp=title, abstract, heading word, drug trade name, original title, device manufacturer, drug manufacturer, device trade name, keyword heading word, floating subheading word, candidate term word]
4. 1 or 2 or 3
5. exp major depression/
6. depression/
7. exp seasonal affective disorder/
8. exp dysthymia/
9. emotional disorder/
10. ((depress* or affective or dysthym*) and (disorder* or disease* or symptom*)).mp. [mp=title, abstract, heading word, drug trade name, original title, device manufacturer, drug manufacturer, device trade name, keyword heading word, floating subheading word, candidate term word]
11. 5 or 6 or 7 or 8 or 9 or 10
12. 4 and 11
13. Randomized controlled trial/ or Controlled clinical trial/ or trial.ti.
14. (random* or blind* or placebo* or meta-analys*).mp. [mp=title, abstract, heading word, drug trade name, original title, device manufacturer, drug manufacturer, device trade name, keyword heading word, floating subheading word, candidate term word]
15. 12 and (13 or 14)
16. limit 15 to (adult <18 to 64 years> or aged <65+ years>)

LILACS (Bireme; 1982 to 7 March 2024) (50 hits)
((mh:(venlafaxine hydrochloride OR d02.033.415.510.500.901 OR d02.092.471.683.948 OR d02.455.426.392.368.367.318.750 OR d10.289.510.500.901 OR mirtazapine OR d03.633.300.240.588)) OR ((venlafaxin* OR ef*exor* OR mirtazapin* OR org*3770 OR remeron*))) AND ((mh:(depressive disorder, major OR f03.600.300.375 OR depressive disorder OR f03.600.300 OR seasonal affective disorder OR f03.600.300.775 OR dysthymic disorder OR f03.600.300.400 OR depression OR f01.145.126.350 OR f01.470.282 OR affective symptoms OR f01.145.126.100)) OR (((depress* OR affective OR dysthym*) AND (disorder* OR disease* OR symptom*)))) AND (db:("LILACS"))

PsycINFO (EBSCO host; 1806 to 7 March 2024) (562 hits)
S17 S15 AND S16
S16 TI adult* or Elder* or older or Geriatri* or Senil* or Old Age* or Late Life or Aged OR AB adult* or Elder* or older or Geriatri* or Senil* or Old Age* or Late Life or Aged
S15 S13 AND S14
S14 TX ((random* or blind* or placebo* or meta-analys*)) OR TI trial*
S13 S4 AND S12
S12 S5 OR S6 OR S7 OR S8 OR S9 OR S10 OR S11
S11 TX ((depress* or affective or dysthym*) and (disorder* or disease* or symptom*))
S10 MA Affective Symptoms
S9 MA Depression
S8 MA Dysthymic Disorder
S7 MA Seasonal Affective Disorder
S6 MA Depressive Disorder Expanders
S5 MA Depressive Disorder, Major
S4 S1 OR S2 OR S3
S3 TX (venlafaxin* or effexor* or efexor* or mirtazapin* or "org 3770" or org3770 or org-3770 or remeron*)
S2 MA mirtazapine
S1 MA venlafaxine

Science Citation Index Expanded (Web of Science; 1900 to 7 March 2024); Conference Proceedings Citation Index – Science (Web of Science; 1990 to 7 March 2024); Social Sciences Citation Index (Web of Science; 1956 to 7 March

Skriv tekst her

2024), and Conference Proceedings Citation Index- Social Science & Humanities (Web of Science; 1990 to 7 March 2024) (653 hits)
#7 #5 AND #6
#6 TS=(adult* or Elder* or older or Geriatri* or Senil* or Old Age* or Late Life or Aged)
#5 #3 AND #4
#4 TI=(random* or blind* or placebo* or meta-analys* or trial*) OR TS=(random* or blind* or placebo* or meta-analys*)
#3 #2 AND #1
#2 TS=((depress* or affective or dysthym*) and (disorder* or disease* or symptom*))
#1 TS=(venlafaxin* or ef*exor* or mirtazapin* or org*3770 or remeron*)

Supplementary Text 3: Searches for unpublished data

**FDA**	**26.09.2024**		
**Search terms**	**Hits**	**Results**	**Comments**
Mirtazapine	1	-	
Remeron	1	FDA review included	
Remeron Soltab	1	-	
Zispin	0	-	
Mirtanza	0	-	
Axit	0	-	
Avansa	0	-	
Combar	0	-	
Mirtin	0	-	
Mirtazapin	0	-	
Zaritim	0	-	
Norset	0	-	
Remergil	0	-	
Remergon	0	-	
Rexer	0	-	
**EMA**	**26.09.2024**		
**Search terms**	**Hits**	**Results**	**Comments**
Mirtazapine	5	-	
Remeron	1	-	
Remeron Soltab	0	-	
Zispin	0	-	
Mirtanza	0	-	
Axit	0	-	
Avansa	0	-	
Combar	0	-	
Mirtin	0	-	
Mirtazapin	0	-	
Zaritim	0	-	
Norset	0	-	
Remergil	0	-	
Remergon	4	-	
Rexer	0	-	

**Japan**	**26.09.2024**		
**Search terms**	**Hits**	**Results**	**Comments**
Mirtazapine	5	-	
Remeron	1	-	
Remeron Soltab	0	-	
Zispin	0	-	
Mirtanza	0	-	
Axit	0	-	
Avansa	0	-	
Combar	0	-	
Mirtin	0	-	
Mirtazapin	0	-	
Zaritim	0	-	
Norset	0	-	
Remergil	0	-	
Remergon	4	-	
Rexer	0	-	
**MHRA**	**26-27.09.2024**		
**Search terms**	**Hits**	**Results**	**Comments**
Mirtazapine	25	-	Using [par] tag - without [par] = 734 hits
Remeron	15	-	
Remeron Soltab	9	-	
Zispin	23	-	
Mirtanza	0	-	
Axit	151	-	Using [par] tag - without [par] = 1097 hits
Avansa	0	-	
Combar	94	-	
Mirtin	74	-	
Mirtazapin	25	-	Using [par] tag - without [par] = 705 hits
Zaritim	0	-	
Norset	11	-	
Remergil	10	-	
Remergon	0	-	

Rexer	3	-	Seached "rexer" (with qoutation marks) because of >11.000 hits without [par] tag and >5000 hits with [par] tag
**TGA ARTG**	**27.09.2024**		
**Search terms**	**Hits**	**Results**	**Comments**
Mirtazapine	46	-	
Remeron	0	-	
Remeron Soltab	0	-	
Zispin	0	-	
Mirtanza	6	-	
Axit	3	-	
Avansa	0	-	
Combar	0	-	
Mirtin	0	-	
Mirtazapin	46	-	
Zaritim	0	-	
Norset	0	-	
Remergil	0	-	
Remergon	0	-	
Rexer	0	-	
**TGA AusPAR**	**27.09.2024**		
**Search terms**	**Hits**	**Results**	**Comments**
Mirtazapine	0	-	
Remeron	0	-	
Remeron Soltab	0	-	
Zispin	0	-	
Mirtanza	0	-	
Axit	0	-	
Avansa	0	-	
Combar	0	-	
Mirtin	0	-	
Mirtazapin	0	-	

Zaritim	0	-	
Norset	0	-	
Remergil	0	-	
Remergon	0	-	
Rexer	0	-	
**CBG MEB (NL)**	**27.09.2024**		
**Search terms**	**Hits**	**Results**	**Comments**
Mirtazapine	55	-	*Translated with Google Translate camera*
Remeron	6	-	
Remeron Soltab	3	-	
Zispin	0	-	
Mirtanza	0	-	
Axit	36	-	
Avansa	0	-	
Combar	0	-	
Mirtin	0	-	
Mirtazapin	55	-	
Zaritim	0	-	
Norset	0	-	
Remergil	0	-	
Remergon	0	-	
Rexer	0	-	
**DPD Canada**	**29-30.09.2024**		
**Search terms**	**Hits**	**Results**	**Comments**
Mirtazapine	60	-	
Remeron	4	-	
Remeron Soltab	0	-	
Zispin	0	-	
Mirtanza	0	-	
Axit	2	-	
Avansa	0	-	
Combar	0	-	
Mirtin	0	-	

Mirtazapin	60	-	
Zaritim	0	-	
Norset	0	-	
Remergil	0	-	
Remergon	0	-	
Rexer	0	-	

On 20 February 2024, we requested clinical study reports via email from the following national medicines agencies:

- U.S. Food and Drug Administration
- European Medicines Agency
- Australia
- Austria
- Belgium
- Bulgaria
- China
- Croatia
- Cyprus
- Czechia
- Denmark
- Estonia
- Finland
- France
- Germany
- Greece
- Hungary
- Iceland
- India
- Ireland
- Italy
- Japan
- Latvia
- Liechtenstein
- Lithuania
- Luxembourg
- Malta
- Netherlands
- Norway
- Poland
- Portugal
- Romania
- Slovakia
- Slovenia

- Spain
- Sweden
- United Kingdom

Supplementary Table 1

Supplementary Table 1: Characteristics of the included trials

Trial ID	Registry/ published protocol	Risk of for-profit bias	Inclusion criteria	Exclusion criteria	Dose range (mg/day)	Control intervention	Placebo washout	Length of intervention period	No. randomised to mirtazapine	No. randomised to control	Baseline HDRS mirtazapine	Baseline HDRS control	Co-interventions
Blier 2010	No	Yes/unclear	For inclusion in the study, patients had to meet DSM-IV criteria for major depressive disorder as a primary diagnosis and had to have a score of at least 18 on the first 17 items of the Hamilton Depression Rating Scale (HAMD-19).	Exclusion criteria included nonresponse to fluoxetine in the index episode, an unstable medical condition, a history of a seizure disorder, abnormal and clinically significant values in the blood workup, and a history of hypomania or mania.	30mg/day	Placebo	No	6 weeks	Unclear	Unclear	22.4	22.6	Yes
Bremner 1995	No	Yes/unclear	Outpatients of both sexes at least 18-years-old with a DSM-III diagnosis of a moderate-to-severe major depressive episode (296.2 or 296.3) and total score ≥ 18 on the first 17 items of the Hamilton Rating Scale for Depression (HAM-D) who were assessed as able to complete the Zung Self-Rating Depression Scale (SDS) entered the study. A fixed upper age limit was not incorporated in the inclusion criteria for this study.	Patients and control volunteers were excluded from the study if they required other psychotropic medications, opiate analgesics, adrenergic agonists or antagonists. A patient could not have received electroconvulsive therapy or monoamine oxidase inhibitors for 2 weeks or tricyclic antidepressants for 3 days prior to the investigation. A urine drug screen was utilized to determine the reliability of the patient drug history and to exclude patients with positive results for abused drugs including alcohol.	22mg/day	Placebo	Yes	6 weeks	Unclear	Unclear	28.3	26.6	No
Carpenter 2002	No	Yes/unclear	Adult outpatients were invited to participate if they met DSM-IV (American Psychiatric Association 1994) criteria for a current major depressive episode and had significant persistent depressive symptoms (total score > 12 on the 17-item Hamilton Rating Scale for Depression [HRSD-17] (Hamilton 1960)) despite at least 4 weeks of standard antidepressant monotherapy at maximum recommended or tolerated doses. Primary antidepressant agents were continued at their prestudy doses throughout the augmentation trial, and introduction of benzodiazepines or other psychotropic agents was not allowed during the four-week assessment period.	NI	15-30 mg	Placebo	No	4 weeks	11	15	21.9	22.5	Yes
Claghorn 1995	No	Yes/unclear	DSM-III episode of moderate or severe major depressive episode, with a total score of ≥ 18 on the first 17 items of the Hamilton depression rating scale (HAMD) and assessed as able to complete Zung's self-rating depression scale (SDS), entered the study. Severity of depression was rated by investigators as moderate to severe.	Exclusion criteria included the following psychiatric disorders: a primary diagnosis of schizophrenia (atypical depressive type); bipolardisorder; anxiety as primary disorder; adjustment disorder; known suicidal tendencies; known cognitive deficit; and alcohol or drug abuse. Patients with symptoms or histories of the following diseases were also excluded: clinically significant renal, hepatic, respiratory, cardiovascular or cerebrovascular diseases; narrow angle glaucoma; clinically significant prostatic hypertrophy; seizure disorders; known drug allergies or other hypersensitivity reactions to tricyclic antidepressants or related compounds; and patients with a clinically significant abnormal EEG or laboratory findings at the screening examination. Only female patients of nonchildbearing potential (e.g., postmenopausal or surgically sterile) were asked to participate in the study. In addition, the following patients were excluded: patients requiring treatment with concomitant psychotropic medication (including benzodiazepines); patients treated with ECT within 3 months of baseline, with monoamine oxidase inhibitors within 14 days from baseline or with other psychotropic drugs (including antidepressants and benzodiazepines) within 7 days of baseline and patients with a total HAMD score reduction of at least 20% in the 7-day placebo washout period. The only permitted psychotropic drug during the trial was chloral hydrate (500 mg in the evening).	Mean: 16.5 mg/day	Placebo	Yes	6 weeks	45	45	21.5	22.7	No
Halikas 1995	No	Yes/unclear	Patients of both sexes over the age of 55 years, diagnosed as having a major depressive episode (DSM lll 296.2 or 296.3) (APA, 1980) with a total score ≥ 18 on the first 17 items of the Hamilton Depression Rating Scale (HAMD) and assessed as able to complete the Zung Self Rating Depression Scale (SDS) were asked to participate in this study.	Exclusion criteria included primary diagnosis of schizophrenia (atypical depressive type); bipolar disorder; adjustment disorder; anxiety as primary disorder; known active suicidal tendencies; known cognitive deficienciesand alcohol or drug abuse in the last six months. Patients with symptoms or a history of the following diseases were to be excluded as well: relevant renal, hepatic, respiratory, cardiovascular or cerebrovascular diseases; narrow angle glaucoma; clinically significant prostatic hypertrophy; seizure disorders; drug allergy or other hypersensitivity reactions to TCA or related compounds; hyperthyroidism; and clinically significant abnormal ECG. Moreover, patients requiring concomitant treatment with other psychotropic medication including benzodiazepines, who had been treated with ECT within 3 months of baseline, who had used MAOIs within 14 days of baseline or other psychotropic drugs including other antidepressants within 7 days of baseline were not acceptable for the study. Finally, patients with a total HAMD score reduction of 20% or more in the 7-day placebo wash-out period were considered to be rapid placebo-responders, and excluded from participation in the study.	20.1 (overall mean including titration period)	Placebo	Yes	6 weeks	Unclear	Unclear	24.6	23.5	No
Jacobson 1990	No	Yes/unclear	All participating patients had a DSM-III diagnosis of major depressive episode (single or recurrent), and a baseline 17-item HAMD score of &18 (Bremner, 1995; Claghorn and Lesem, 1995; Jacobson et al., 1990; Smith et al., 1990; Data on ®le, NV Organon) or a baseline score on the 21-item HAMD of&18 (Vartiainen and Leinonen, 1994; Data on ®le, NV Organon). The duration of the present depressive episode was between 14 d and 6 months, and patients had less than 6 depressive episodes requiring hospitalization in the past.	Excluded from the studies were patients with more than a 25% decrease in total HAMD score during the placebo washout period ; those with a history of schizophrenia or other psychoses, atypical depression, adjustment disorder, drug or alcohol abuse; drug overdose in the previous 4 months; and active suicidal tendencies. Patients with clinically relevant renal, cardiovascular, respiratory or cerebrovascular diseases, prostatic hypertrophy, narrow angle glaucoma, urinary retention, unstable diabetes, seizure disorder or clinically relevant EEG changes were also ineligible for participation in the studies. None of the patients had been treated with ECT in the previous 3 months, or with an adequate dose of an antidepressant (&150 mg amitriptyline or equivalent for at least 6 wk) in the month preceding the trial. Women of child-bearing potential had to be adequately protected against pregnancy, while mothers, either breastfeeding or within 6 months post-partum, were excluded from the studies.	Mean dose last week: 20.2 mg	Placebo	Yes	4 or 6 weeks	Unclear	Unclear	21	21.4	No
Kessler 2018	Yes	Yes/unclear	Aged ≥ 18 years and in primary care. Treated for depression for at least 6 weeks with any one of the following SSRI or SNRI antidepressants at recommended BNF doses – fluoxetine, sertraline, citalopram, escitalopram, fluvoxamine, paroxetine, duloxetine or venlafaxine (see Appendix 1, Table 25 for the adequate dose table). Adhered to their medication. Adherence to medication is difficult to measure. To operationalise our definition of treatment resistance, we used the Morisky four-item self-report measure of compliance, as adapted for CoBalT. The Morisky measure has previously been validated against electronic monitoring bottles, with a score of zero (range 0–4) indicating at least 80% compliance. Given the relatively long half-life of antidepressant medication, individuals who have forgotten to take one or two tablets were not excluded. Scored ≥ 14 points on the Beck Depression Inventory-II (BDI-II). An ICD-10 diagnosis of depression [assessed using the Clinical Interview Schedule – Revised (CIS-R)].	Patients currently taking combined or augmented antidepressant treatment. Patients having their medication managed by a psychiatrist - patients with dementia (formal diagnosis), bipolar disorder, psychosis or alcohol or substance abuse/dependence - women who were pregnant, planning a pregnancy or breastfeeding - patients who were unable to complete the study questionnaires - patients who had had a previous adverse reaction to mirtazapine - patients currently being treated with a monoamine oxidase inhibitor (MAOI), including moclobemide, or with other medical contraindications to mirtazapine.	15-30mg/day	Placebo	No	52 weeks	241	239	31.5	30.6	Yes
Kinoshita 2009 (15 mg)	No	Yes/unclear	Study inclusion criteria were: (1) aged 20–75 years old; (2) primary diagnosis of MDD according to the Diagnostic and Statistical Manual of Mental Disorders, Fourth Edition (DSM-IV)17; (3) a baseline total score of 18 or higher on the HAM-D17; and (4) capability of providing informed consent.	The patients with more than 25% decrease in total HPRSD score during the placebo-washout period; those with a history of schizophrenia (atypical depressive type) or other psychoses, anxiety disorder or adjustment disorder, drug or alcohol abuse in the last 2 years, or active suicidal tendencies were not eligible for participation in the study. Patients with clinically relevant renal, cardiovascular (except for mild, stable hypertension), respiratory or cerebrovascular diseases, prostatic hypertrophy, narrow angle glaucoma, urinary retention, unstable diabetes, seizure disorder or clinically relevant ECG abnormalities were not eligible as well. At baseline, the patients could have a maximum of 3 abnormal laboratory variables. None of the patients had been treated with ECT, or with an adequate dose of an antidepressant (2 150 mg amitriptyline or equivalent for at least 6 weeks) in a month preceding the trial. Concomitant use of other psychotropic medication was prohibited, except for chloral hydrate (max. 3000 mg during the whole study period) or, if unavoidable, shortacting benzodiazepines (for example lorazepam, oxazepam, triazolam) for night time sedation during the first 14 days of the study only. Women of child-bearing potential had to be adequately protected against pregnancy, while mothers, either breast-feeding or within 6 months post-partum, were excluded from the study.	15 mg/day	Placebo	No	6 weeks	70	24	23.2	22.5	No
Kinoshita 2009 (30 mg)	No	Yes/unclear	As above	As above	30 mg/day	Placebo	No	6 weeks	70	23	22.5	22.5	No
Kinoshita 2009 (45 mg)	No	Yes/unclear	As above	As above	45 mg/day	Placebo	No	6 weeks	71	23	22.1	22.5	No
Organon 3-020	No	Yes/unclear	All participating patients had a DSM-III diagnosis of major depressive episode (single or recurrent), and a baseline 17-item HAMD score of ≥18 (Bremner, 1995; Claghorn and Lesem, 1995; Jacobson et al., 1990; Smith et al., 1990; Data on file, NV Organon) or a baseline score on the 21-item HAMD of&18 (Vartiainen and Leinonen, 1994; Data on file, NV Organon). The duration of the present depressive episode was between 14 d and 6 months, and patients had less than 6 depressive episodes requiring hospitalization in the past.	Excluded from the studies were patients with more than a 25% decrease in total HAMD score during the placebo washout period ; those with a history of schizophrenia or other psychoses, atypical depression, adjustment disorder, drug or alcohol abuse; drug overdose in the previous 4 months; and active suicidal tendencies. Patients with clinically relevant renal, cardiovascular, respiratory or cerebrovascular diseases, prostatic hypertrophy, narrow angle glaucoma, urinary retention, unstable diabetes, seizure disorder or clinically relevant EEG changes were also ineligible for participation in the studies. None of the patients had been treated with ECT in the previous 3 months, or with an adequate dose of an antidepressant (≥ 150 mg amitriptyline or equivalent for at least 6 wk) in the month preceding the trial. Women of child-bearing potential had to be adequately protected against pregnancy, while mothers, either breastfeeding or within 6 months post-partum, were excluded from the studies.	Mean dose last week: 22.5 mg	Placebo	Yes	6 weeks	Unclear	Unclear	23.8	25.2	No
Organon UK unpublished	No	Yes/unclear	All participating patients had a DSM-III diagnosis of major depressive episode (single or recurrent), and a baseline 17-item HAMD score of &18 (Bremner, 1995; Claghorn and Lesem, 1995; Jacobson et al., 1990; Smith et al., 1990; Data on file, NV Organon) or a baseline score on the 21-item HAMD of&18 (Vartiainen and Leinonen, 1994; Data on file, NV Organon). The duration of the present depressive episode was between 14 d and 6 months, and patients had less than 6 depressive episodes requiring hospitalization in the past.	Excluded from the studies were patients with more than a 25% decrease in total HAMD score during the placebo washout period ; those with a history of schizophrenia or other psychoses, atypical depression, adjustment disorder, drug or alcohol abuse; drug overdose in the previous 4 months; and active suicidal tendencies. Patients with clinically relevant renal, cardiovascular, respiratory or cerebrovascular diseases, prostatic hypertrophy, narrow angle glaucoma, urinary retention, unstable diabetes, seizure disorder or clinically relevant EEG changes were also ineligible for participation in the studies. None of the patients had been treated with ECT in the previous 3 months, or with an adequate dose of an antidepressant (≥ 150 mg amitriptyline or equivalent for at least 6 wk) in the month preceding the trial. Women of child-bearing potential had to be adequately protected against pregnancy, while mothers, either breastfeeding or within 6 months post-partum, were excluded from the studies.	Mean dose last week: 52.5 mg	Placebo	Yes	5 weeks	Unclear	Unclear	23.3	22.9	No
Rao 2019	No	No	Current Major Depressive Disorder (MDD) DSM- IV diagnosis, confirmed by a structured diagnostic interview by a psychology therapist with 17 Item-HDRS score≥18, •Age between 18 and 65 years, •On escitalopram only for more than or equal to 4 weeks, •No associated co-morbid conditions(e.g., hypertension, diabetes).	Patients with psychosis or delusional disorders (Current or past), acute suicidal tendency, mental retardation, patientshaving a history of current substance abuse or dependence, a serious and unstable medical condition (e.g., pacemaker,porphyria) and history of epileptic seizures had been excluded from the study. Pregnant and lactating mothers were not eligible for the study.	7.5 mg/day	Placebo	No	6 weeks	30	30	23.2	23.8	Yes

Supplementary Table 1

Remeron report 2334 (15 mg)	No	Yes/unclear	Male or female aged 18-70 years - probable diagnosis of endogenous depression - a diagnosis of major depression according to DSM III and the Research Diagnostic Criteria (RDC) moderate to severe depression with a score > 18 on a 21-item Hamilton Psychiatric Rating Scale for Depression (HPRSD) not responding to placebo treatment during the 4-9 day washout period as assessed by a decrease of > 25% in the total HPRSD score - suffering from depression for at least 14 days and not more than 6 months not more than 3 previous episodes of depression which required hospitalization - being adequately protected against pregnancy, not pregnant or nursing mothers - giving informed consent - no history of schizophrenia or other psychosis or adjustment or atypical depressive disorder - not known to have significant suicidal tendencies - not having more than 3 clinically relevant, abnormal laboratory parameters at baseline - not having clinically relevant renal, hepatic, cardiovascular or cerebro-vascular disease - not having prostatic hypertrophy, narrow angle glaucoma or seizure disorder - no drug or alcohol abuse or having had a drug overdose in the previous 4 months - not having unstable diabetes - not having clinically relevant ECG abnormalities - not having received ECT in the previous 3 months - not having received treatment with an adequate dose of an antidepressant in the previous month<br>- not requiring concomitant therapy with CNS active drugs with the exception of short-acting benzodiazepines for night sedation.	NI	15 mg/day	Placebo	Yes	5 weeks	13	4	NI	NI	No
Remeron report 2334 (30 mg)	No	Yes/unclear	As above	NI	15-30 mg/day	Placebo	Yes	5 weeks	13	4	NI	NI	No
Remeron report 2334 (60 mg)	No	Yes/unclear	As above	NI	15-60 mg/day	Placebo	Yes	5 weeks	13	4	NI	NI	No
Smith 1990	No	Yes/unclear	The study population consisted of 150 outpatients with a diagnosis of major depressive illness, DSM-lll 296.2 or 296.3 (American Psychiatric Association 1980) and a minimum baseline score of 18 on the first 17 items of the Hamilton Rating Scale for Depression (HAM-D; Hamilton 1960). Patients who had a 20 percent or greater reduction in total HAM-D score during the placebo washout period were considered placebo responders and were not randomized into the study. Additionally, patients were required to be at least 18 years of age; free of significant renal, hepatic, respiratory, cardiovascular, or cerebrovascular disease; free of narrow angle glaucoma, prostatic hypertrophy, and seizure disorders; and with no clinically relevant abnormal laboratory values or significantly abnormal electrocardiogram (EKG) findings.	Furthermore, patients were excluded if their primary diagnosis was schizophrenia, atypical depression, anxiety, adjustment, or bipolar disorder, or if they were known drug or alcohol abusers or had known active suicidal tendencies or known cognitive deficiencies.	Max: 35 mg	Placebo	Yes	6 weeks	50	50	23.4	23.3	No
Vartiainen 1994	No	Yes/unclear	All patients suffered from major depressive episode (single or recurrent) according to DSM-III criteria (APA, 1980) and RDC (Spitzer et al., 1978) and had a total score of 18 or more on a 21-item Hamilton Depression Scale (HAM-D, Hamilton, 1960) at screening and baseline.	Patients with psychiatric complaints other than primary major depression, and those with clinically relevant physical illness were excluded from the study.	32,3 (mean dose at day 42)	Placebo	Yes	6 weeks	Unclear	Unclear	25.6	25.9	No
Xiao 2021	Yes	Yes/unclear	Inclusion criteria: Male or female outpatients, aged 18–60 years (inclusive), with a diagnosis of a major depressive episode (single or recurrent) according to DSM-IV criteria. Participants were required to have a total HAMD-17 score ≥ 20, and score ≥ 2 on item 1 (depressed mood) at enrolment in phase 1.	Exclusion criteria: Use of antipsychotics or mood stabilizers within the 5 days prior to screening. Use of depot antipsychotic medication within one cycle prior to screening. Known allergy or lack of response to mirtazapine and paroxetine. Participants receiving an investigational agent (including different formulations and generic agents of the investigational drug) within 3 months prior to screening. A current Axis I primary psychiatric diagnosis other than MDD. Lifetime history of alcohol abuse or dependence. Organic mental disease, including mental retardation. Current or recent (discontinued within the last 30 days) enrolment in a clinical trial involving an off-label use of an investigational drug. History of any cardiovascular, hepatic, renal, respiratory, haematological, endocrinological or neurological disease, or clinically significant laboratory abnormality that is not stabilized or is anticipated to require treatment during the study. Pregnant or lactating women, or women of child bearing potential without appropriate birth control measures. Previous treatment with ECT or MECT within 3 months prior to screening. Significant risk of suicidal and/or self-harm behaviours. Any systematic psychotherapy (psychoanalysis, cognitive comprehension, desensitization therapy, hypnosis therapy, Morita therapy) was prohibited.	30mg/day	Placebo	No	6 weeks	68	68	20.92	21.64	Yes
003-003	NI	Yes/unclear	A diagnosis of Major Depressive Disorder (DSM III criteria)<br>Score on the HAM D-17 of at least 18 on day 0.<br>Ages between 18 and 65 years old (females either surgically sterile or postmenopausal)<br>Good physical health and no significant medical history and abnormal laboratory values.	A history of a primary diagnosis of<br>• schizophrenia or other psychosis.<br>• anxiety disorder<br>• adjustment disorder<br>• dysthymic or cyclothymic disorder<br>• atypical depressive disorder.<br>• known suicidal tendency.<br>-Heavy smokers (15 cigarettes or equivalent per day) or users of alcohol during the study.<br>-Treatment with ECT within 3 months of day o.<br>-Patients on neuroleptics, MAO inhibitors, other anti-depressants, stimulants, appetite suppressors or other psychotropic drugs within seven days of day O.<br>-Concomitant therapy with any CNS active drug, except for a nighttime sedative (1 gram of chloral hydrate for night-time sedation).	26.6 mg/day (mean dose in week 6)	Placebo	NI	6 weeks	45	45	NI	NI	No
003-008	NI	Yes/unclear	A diagnosis of Major Depressive Disorder (DSM III criteria)<br>Score on the HAM D-17 of at least 18 on day 0.<br>Ages between 18 and 65 years old (females either surgically sterile or postmenopausal)<br>Good physical health and no significant medical history and abnormal laboratory values.	A history of a primary diagnosis of<br>• schizophrenia or other psychosis.<br>• anxiety disorder<br>• adjustment disorder<br>• dysthymic or cyclothymic disorder<br>• atypical depressive disorder.<br>• known suicidal tendency.<br>-Heavy smokers (15 cigarettes or equivalent per day) or users of alcohol during the study.<br>-Treatment with ECT within 3 months of day o.<br>-Patients on neuroleptics, MAO inhibitors, other anti-depressants, stimulants, appetite suppressors or other psychotropic drugs within seven days of day O.<br>-Concomitant therapy with any CNS active drug, except for a nighttime sedative (1 gram of chloral hydrate for night-time sedation).	5 - 60mg/day	Placebo	NI	6 weeks	120	30	NI	NI	No

Supplementary Table 2: Summary of findings

Mirtazapine compared to control for major depressive disorder

Patient or population: major depressive disorder

Setting:

Intervention: mirtazapine

Comparison: control

Outcomes	Anticipated absolute effects* (95% CI)		Relative effect (95% CI)	№ of participants (studies)	Certainty of the evidence (GRADE)	Comments
	Risk with control	Risk with mirtazapine				
Suicide or suicide attempts follow-up: range 5 weeks to 6 weeks	7 per 1.000	**13 per 1.000** (2 to 70)	**OR 1.99** (0.36 to 11.07)	299 (3 RCTs)	⨁◯◯◯ Very low[a,b]	
Serious adverse events follow-up: range 4 weeks to 12 weeks	18 per 1.000	**32 per 1.000** (17 to 59)	**OR 1.82** (0.95 to 3.48)	1439 (11 RCTs)	⨁◯◯◯ Very low[a,b,c]	
Non-serious adverse events follow-up: range 4 weeks to 12 weeks	159 per 1.000	**376 per 1.000** (241 to 588)	**RR 2.36** (1.51 to 3.69)	1477 (11 RCTs)	⨁◯◯◯ Very low[a,b,c]	

***The risk in the intervention group** (and its 95% confidence interval) is based on the assumed risk in the comparison group and the **relative effect** of the intervention (and its 95% CI).

CI: confidence interval; **OR:** odds ratio; **RR:** risk ratio

GRADE Working Group grades of evidence
High certainty: we are very confident that the true effect lies close to that of the estimate of the effect.
Moderate certainty: we are moderately confident in the effect estimate: the true effect is likely to be close to the estimate of the effect, but there is a possibility that it is substantially different.
Low certainty: our confidence in the effect estimate is limited: the true effect may be substantially different from the estimate of the effect.
Very low certainty: we have very little confidence in the effect estimate: the true effect is likely to be substantially different from the estimate of effect.

Explanations

a. Downgraded 2 levels for high risk of bias in the included studies.

b. Downgraded 2 levels for imprecision due to Trial Sequential Analysis showing that there was not enough information to confirm or reject a relative risk reduction (RRR) of 20% and the accrued number of participants is below 50% of the diversity-adjusted required information size (DARIS).

c. Downgraded 1 level for indirectness due to differences in measurement of outcome.

Supplementary table 3: Serious adverse events in the included trials				
Trial ID	Mirtazapine group		Control group	
	Numbers and types of serious adverse events	Proportion of participants with a serious adverse event	Numbers and types of serious adverse events	Proportion of participants with a serious adverse event
Blier 2010	2 anxiety	2 out of 25	-	0 out of 28
Carpenter 2002	2 ringing in ears/trouble hearing, 1 hearing/seeing things	* out of 11	2 sexual arousal problems	* out of 15
Halikas 1995	-	0 out of 50	1 urinary retention	1 out of 50
Kessler 2018	1 fall, 1 gynaecological procedure, 1 DVT, 1 transient ischaemic attack, 1 dental extraction, 1 suicidal ideation and self-harm, 1 deliberate overdose, 1 pancreatitis	* out of 241	2 fall, 1 infective gastroenteritis	* out of 239
Organon UK unpublished	1 thyroglossal cyst, 1 suicide attempt, 1 temporary partial loss of some psychomotor functions, 1 parasuicide	* out of 66	1 suicide attempt, 1 erythematous rash, 1 loss of all psychomotor activity	* out of 66
Smith 1990	3 hypertension, 2 hypotension	* out of 50	3 hypertension, 2 hypotension	* out of 50
Vartiaien 1994	2 suicides	2 out of 59	-	0 out of 55
003-003	1 decline of white blood cells	1 out of 45	-	0 out of 45
003-008	-	0 out of 120	-	0 out of 30
*Proportion unclear.				

Supplementary Table 4: Individual non-serious adverse events								
**Events**	**Number of trials reporting the event**	**Mirtazapine events**	**Mirtazapine analysed**	**Control events**	**Control analysed**	**Relative risk (95% CI)**	**P-value**	**Number needed to harm (NNH)/treat (NNT)**
Somnolence	10	264	814	67	674	2.61 (1.26, 5.37)	0.01	NNH: 5
Weight increased	7	75	611	12	476	4.75 (2.05, 10.99)	< 0.01	NNH: 10
Dry mouth	9	134	557	65	419	1.57 (1.02, 2.42)	0.04	NNH: 11
Dizziness	6	40	467	11	325	2.54 (1.18, 5.45)	0.02	NNH: 19
Headache	8	38	666	55	531	0.54(0.35, 0.83)	< 0.01	NNT: 22
Increased appetite	7	33	625	7	485	3.22 (1.34, 7.73)	0.01	NNH: 26
Amblyopia	3	6	150	2	150	1.90 (0.33, 10.99)	0.47	
Asthenia	5	18	199	15	202	1.24 (0.51, 3.02)	0.63	
Blurred vision	2	2	75	2	74	1.17 (0.12, 11.58)	0.89	
Constipation	8	55	503	21	362	1.49 (0.62, 3.57)	0.38	
Dyspepsia	3	1	100	1	100	0.52 (0.11, 2.54)	0.42	
Insomnia	2	5	54	7	57	0.74 (0.24, 2.26)	0.60	
Nausea	5	9	393	17	394	0.58 (0.20, 1.69)	0.32	
Sedation	2	13	89	5	87	2.61 (0.96, 7.05)	0.06	
Stomach discomfort	2	9	221	7	85	0.57 (0.15, 2.22)	0.42	
Tachycardia	2	2	100	3	100	0.67 (0.11, 4.07)	0.67	
Tremor	4	2	111	3	115	0.64 (0.14, 3.02)	0.57	
